# Investigating the role of abscisic acid and its catabolites on senescence processes in green asparagus under controlled atmosphere (CA) storage regimes

**DOI:** 10.1016/j.postharvbio.2022.111892

**Published:** 2022-06

**Authors:** Maria Anastasiadi, Emma R. Collings, Leon A. Terry

**Affiliations:** a Plant Science Laboratory, Cranfield University, Bedfordshire MK43 0AL, UK; b Cobrey Farms Ross-on-Wye, Herefordshire HR9 5SG, UK

**Keywords:** *Asparagus officinalis*, Abscisic acid (ABA), Sugars, ABA catabolites

## Abstract

Asparagus (*Asparagus officinalis*) is a highly perishable crop with a short postharvest life. Although some research has been done on the application of controlled atmosphere (CA), it has not been sufficiently explored and the underlying mechanisms controlling asparagus senescence processes are not well understood, restricting its potential for commercial application. The aim of this study was to investigate for the first time the link between abscisic acid (ABA) and ABA catabolites and senescence in asparagus stored under a range of different CA conditions. Two different set-ups were run in parallel; a traditional CA delivered by an International Controlled Atmosphere (ICA) system with continuous gas supply and LabPods™ fitted with sensors for real time monitoring of respiration rate (RR) and respiratory quotient (RQ) and able to retain established CA conditions with minimum gas supply requirements. The role of genetic variability was also studied by including two UK grown asparagus cultivars ‘Gijnlim’ and ‘Jaleo’ adapted for different climatic conditions. The results indicated that ABA and its catabolites were present in significantly higher concentrations in the air stored spears (control) compared to CA throughout storage, irrespective of cultivar, and were associated with accelerated senescence processes observed in control samples, such as textural changes indicative of spear toughening, discolouration, sugar depletion and asparagine accumulation. Furthermore, partial least squares regression (pls-r) applied for both cultivars, successfully differentiated samples based on O_2_ and CO_2_ concentrations and storage duration, both in cold storage and during shelf-life with the separation being driven primarily by ABA and its catabolites. Physiological and biochemical results indicated that all three CA conditions tested ([CA1] 2.5% O_2_, 3% CO_2_, [CA2] 2.5% O_2_, 6% CO_2_ and [CA3] 2.5% O_2_, 10% CO_2_) successfully retained quality parameters including texture, colour, moisture content and visual appearance longer compared to air (control); however, they did not completely suppress the development of ‘tip-breakdown’ (a physiological disorder also known as tip rot) towards the end of storage, which coincided with rising concentrations of phaseic acid indicating an activation of the abscisic biosynthetic and catabolic pathway. It can be concluded that CA conditions can delay senescence for at least 3-weeks (2 weeks cold storage and 1 week shelf-life), by lowering metabolic rate and respiratory quotient (RQ) within the spears compared to control, and through successfully regulating ABA biosynthetic and catabolic pathways.

## Highlights

• ABA and PA were associated with accelerated senescence processes in asparagus.
• CA extended asparagus storage life by suppressing ABA and ABA catabolite accumulation.
• Low O_2_ and high CO_2_ helped maintain higher sugar content compared to control.
• LabPods™ enabled real-time monitoring of asparagus metabolic rates.

## 1 Introduction

Asparagus is a highly perishable seasonal vegetable, which has increased in popularity over the past years due to its unique taste and nutritional value. The short UK harvest season for asparagus typically lasts from April to June, which means that the UK market heavily depends on imported asparagus to cover consumer demand during the rest of the year. Asparagus deterioration during air storage is accompanied by rapid sugar loss due to high metabolic rates, undesirable texture changes (lignification), chlorophyll degradation, ascorbic acid and organic acid loss and asparagine accumulation (Irving et al., 2001, Renquist et al., 2005). Furthermore, postharvest physiological disorders including microbial decay and tip breakdown (a physiological disorder, also known as tip rot) further contribute towards quality reduction in asparagus spears, although it is still unclear how the latter is caused (Lallu et al., 2000).

Currently, the most effective method used by the industry to suppress respiration, and thus preserve quality, is to hydro-cool immediately after harvest and maintain storage and transit temperatures between 0 and 2 °C at 90–95% RH to avoid excess water loss. These conditions alone can only typically preserve asparagus spears for up to a week before visual deterioration in quality occurs. Other technologies which have been explored for delaying the senescent processes in asparagus include the application of controlled atmosphere (CA) (O_2_: 2–5 kPa and CO_2_: 10–15 kPa) (Lill and Corrigan, 1996, Hurst et al., 1996, Renquist et al., 2005) and modified atmosphere packaging (MAP) (O_2_: 5–16 kPa and CO_2_: 5–14 kPa) (Tenorio et al., 2004, Villanueva et al., 2005, Zhang et al., 2008). Despite these studies, CA and MAP have not been extensively used commercially possibly due to insufficient benefits compared to cost. Anecdotal evidence has also indicated that CA decreases shelf-life (personal communication, Cobrey Farms). Therefore, additional work is required to explore in greater detail the underlying mechanisms involved during asparagus senescence under CA or MAP conditions.

It is well known that abscisic acid (ABA) plays a central role (along with ethylene) in regulating leaf senescence. Suppression of ABA synthesis reportedly delays senescence in a variety of plants including Chinese flowering cabbage, tobacco leaves and ryegrass (Zhang et al., 2017, Tan et al., 2019). Furthermore, ABA responsive genes in Arabidopsis activate chlorophyll catabolic enzyme genes (Gao et al., 2016). The role of ABA in asparagus, has recently been found to negatively impact on the quality of asparagus during storage with high levels correlating with low sugar content and reduced shelf life (Anastasiadi et al., 2020). However, there is a paucity of other research exploring hormonal flux during asparagus storage particularly under CA/MAP conditions.

Application of continuous respiratory quotient (RQ) monitoring as a way to detect metabolic changes postharvest has been employed commercially for apples leading to improved storage life (Weber et al., 2015), but to our knowledge, this has not been explored for asparagus. At harvest, the loss of carbohydrate supply and the continuing cellular respiration, leads to sugar depletion particular in asparagus tips which are comprised of highly active meristematic tissues. In these tissues, respiration rate (RR) has been reported to be up to 4 times higher during cold storage in asparagus acropetal regions compared to the basal sections, and thus is more susceptible to senescent deterioration (Verlinden et al., 2014). Determining RQ *in situ* during storage of asparagus could enable continuous monitoring of metabolic activity leading to a better understanding of the biochemical changes and senescent processes taking place under air and CA conditions.

In this work, various CA environments consisting of low O_2_ combined with high CO_2_, were explored during asparagus cold storage. LabPods™ were also employed to continuously record RR and RQ during cold storage in an effort to improve our understanding of how different gas combinations influence asparagus metabolic rates *in situ* and subsequent changes to physiology and biochemical profile. Changes in the plant hormone ABA and its catabolites were for the first time, measured in asparagus during CA storage enabling an insight into senescence processes.

## 2 Materials and methods

### 2.1 Comparing air storage with different CA conditions

#### 2.1.1 Plant material

The first two experiments were performed on two successive years on UK grown asparagus spears cultivar ‘Gijnlim’ harvested from 13-year-old crowns. Asparagus were grown in the UK in sandy soil under commercial growing conditions. Spears were hand-cut at ground level between 0730 and 0900 h at relative field temperatures of 12 ± 4 °C and hydro-cooled within 2 h from harvest. Medium graded spears of *ca*. 15 mm diameter were stored overnight at 1 °C before transportation to Plant Science Laboratory within 3 h by refrigerated (at 4 °C) transport.

#### 2.1.2 Experimental design

Upon arrival at the laboratory, a random sub-sample of 18 spears were selected for physiological and biochemical analysis. The rest of the spears were randomly divided into Lock & Lock™ (12 L) plastic storage boxes. Across the two years, three CA conditions were explored in comparison to control comprised of low O_2_ concentrations and elevated CO_2_ concentrations. The specific gas concentrations for each CA regime were as follows: [**CA1**] - 2.5 kPa O_2_ + 3 kPa CO_2_; [**CA2**] - 2.5 kPa O_2_ + 6 kPa CO_2_; [**CA3**] - 2.5 kPa O_2_ + 10 kPa CO_2._ Table 1 lists the controlled atmosphere conditions selected for each year. A sub sample of asparagus received in year 1 were also stored in LabPods™ (see Section 2.2.).

**Table 1:** Overview of three experiments conducted over 2 years on two different cultivars (‘Gijnlim’ and ‘Jaleo’) selected for controlled atmosphere (CA) storage (within either LabPod™ or boxes) under different gas conditions and control (air).

**Year**	**Experiment no.**	**Cultivar**	**Storage type**	**CA**
1	1	‘Gijnlim’	CA (LabPods and Boxes)	Control 2.5 kPa O_2_ + 3 kPa CO_2_ [CA1] 2.5 kPa O_2_ + 6 kPa CO_2_ [CA2]
2	2	‘Gijnlim’	CA (Boxes)	Control 2.5 kPa O_2_ + 6 kPa CO_2_ [CA2] 2.5 kPa O_2_ + 10 kPa CO_2_ [CA3]
2	3	‘Jaleo’	CA (Boxes)	Control 2.5 kPa O_2_ + 6 kPa CO_2_ [CA2]

At each sampling week, three replicate 12 L storage boxes per treatment (CA *vs.* control in air) were opened and then discarded. Gas concentration within the storage boxes was controlled using an ICA gas mixing system (Storage Control Systems Ltd, Kent, UK) as described in Alamar et al. (2017). Boxes were fitted with an inlet and an outlet to allow continuous air or CA gas exchange inside the boxes, preventing a build-up of CO_2_. Tissue paper soaked in deionised water was placed underneath freshness trays inside the boxes to maintain a high RH (*ca.* ≥ 90%). Sampling from the storage boxes was performed every 7 days for four weeks in year 1, and three weeks in year 2. A subset of spears (*ca*. 6 spears) from each box were held under shelf-life conditions at 7 °C in an environmental test chamber (Sanyo MLR-351 H, Osaka, Japan) under artificial light (*ca*. 1358 lx) and 70% RH for up to seven days to simulate retail conditions (**S1**). Shelf-life assessment was performed at the end of storage or after each week for year 1 and 2, respectively.

### 2.2 Real time RR and RQ monitoring using Labpods

As stated in Section 2.1., a sub sample of spears from year 1 were randomly divided into LabPods™ (Storage Control Systems Ltd, Kent UK) with the aim to study the influence of CA conditions on asparagus metabolic rates in real-time. Due to the limited number of LabPods™ available, only CA2 and control were assessed within LabPods™ with two assigned to each treatment. Each LabPod™ was filled with *ca*. 21 Kg of asparagus spears equally distributed into three plastic crates. The setup for LabPods™ is depicted in picture **S2**.

LabPods™ remained undisturbed for the duration of cold storage (4 weeks) replicating a commercial scenario. Ultrasonic humidifiers maintained a high RH inside each LabPod™. Gas concentrations were monitored automatically every hour and adjusted accordingly. In addition, RR and RQ measurements were automatically recorded every 48 h by the LabPod™ system. Once opened, spears were randomly selected for physiological and biochemical analysis, alongside additional spears for shelf-life assessment (in air at 7 °C for up to 7 days in light, as previously described by Anastasiadi et al., 2020).

### 2.3 Assessment of CA storage conditions on a warm acclimated cultivar

#### 2.3.1 Plant material

One of the CA regimes established for ‘Gijnlim’ [CA2 (2.5 kPa O_2_ + 6 kPa CO_2_)] were assessed on another cultivar ‘Jaleo’ which were harvested from 8-year-old crowns grown under the same soil conditions and following the same harvest procedure as detailed in Section 2.1. However, for ‘Jaleo’, which is a warm-adapted Peruvian variety, spears were grown under transparent polythene cloches (100 µm thick Daios thermic pocket polythene) that was removed when temperatures exceeded 30 °C.

#### 2.3.2 Experimental design

Spears of cultivar ‘Jaleo’ were randomly divided into 12 L storage boxes as described in Section 2.1, and stored under CA2 (2.5 kPa O_2_ + 6 kPa CO_2_) and control (air) conditions for four weeks (Table 1). Three replicate boxes per treatment were sampled weekly and a subset of spears (n = 6) were stored under shelf-life conditions as described in Section 2.1.

### 2.4 Physiological assessments

Physiological measurements were performed on spears sampled after cold storage (n = 3 spears per box) and shelf-life (n = 2 spears per tray). Samples were trimmed to 15 cm to simulate commercial practices and marked at 4 cm and 11 cm from the tip (see Section 2.4.2).

#### 2.4.1 Moisture loss

A cluster, containing *ca.* 15 spears, was weighed before and after cold storage within each LabPod™ (two replicates per treatment). For samples stored in 12 L boxes, the total weight of spears placed into each box was recorded and weighed again at the corresponding sampling day (three replicates per treatment). For shelf-life assessment, the weight of spears was recorded for each tray before and after storage at 7 °C (three trays per treatment).

#### 2.4.2 Cutting energy

Cutting energy was measured with a uniaxial testing machine (Instron 5542, Instron, Buckinghamshire, UK) as described in Anastasiadi et al. (2020). Cutting energy (mJ) defined as the force required for cutting the spear at a depth of 2 and 4 mm was used as a measure of firmness at two different positions: apical (4 cm from the tip) and middle (11 cm from the tip).

#### 2.4.3 Objective colour

Objective colour assessments of the tip (0–4 cm from top) and basal regions of the spears (11–15 cm from top) were performed as described in Anastasiadi et al. (2020). Samples were placed inside a Photo-E-Box plus 1419 set at D65 (6500 K) under LED lights (back, left and right) and images of the whole spear were captured using a Lumenera Infinity 3 high definition digital camera with CCD colour sensor (Lumenera Corporation, Ottawa, ON). Objective colour measurements were extracted and processed with the associated Infinity Analyse software version 6.5 to obtain colour parameters (red [R*], green/red axis component [G*] and yellow/blue axis component [B*]) for each selected region (*viz.* tip and base). Areas such as bracts or damaged regions were excluded to avoid skewing the data. The RGB values were later converted into lightness (L*), chroma (C*) and hue angle (h°) using standard RGB conversion equations for D65 (Easy RGB, 2018).

#### 2.4.4 Subjective visual assessment

Upon removal from cold storage and after the end of shelf-life, spears were also visually inspected for the presence of mould and ‘tip breakdown’. The results were expressed in percentage of spears per treatment with mould, or ‘tip breakdown’ and a ‘freshness’ score per treatment was attributed from 0 to 5, with zero corresponding to ‘unacceptable’ and five corresponding to ‘fresh’.

#### 2.4.5 Visual assessment after cooking

Randomly selected spears from each box (*ca*. 6–10 per treatment) were trimmed to 15 cm and boiled in water for 3 min. Spears were visually evaluated by a semi-trained panel to subjectively assess appearance and texture.

### 2.5 Biochemical assessments

#### 2.5.1 Non-structural carbohydrates and L-asparagine

After physiological assessments, samples were immediately snap-frozen in liquid nitrogen and stored at −40 °C, before subsequent freeze drying and powdering in a homogenizer (Precellys 24, Stretton Scientific Ltd, UK) at 5000 rpm for 20 s using ceramic beads. The freeze-dried powder was stored at −40 °C. Non-structural carbohydrates and the amino acid L-asparagine were extracted according to the method described in Anastasiadi et al. (2020).

#### 2.5.2 ABA and catabolites

The seasonal variation in ABA and ABA catabolites was assessed according to the method by Ordaz-Ortiz et al. (2015) with modifications as described in Anastasiadi et al. (2020).

### 2.6 Statistical assessment

Statistical analyses were carried out using Statistica for Windows version 10, 64-bit (Statistica Tulsa, OK 74104, USA). Analysis of variance (ANOVA) was used to identify significant differences (*p* < 0.05) between treatments followed by Fisher’s post-hoc test. Standard errors for each mean are displayed in each applicable Fig and Table. The R package pls was used for partial least squares regression analysis (pls-r). The measured physiological and biochemical parameters were used as predictor variables while O_2_ concentration, CO_2_ concentration and storage time were used as response variables. All the variables were auto scaled prior to analysis. Important variables were selected based on jack-knife-based resampling and only metabolites with stable (*p* < 0.05) regression coefficient were selected to train the pls-r model.

Correlation analysis was performed with the R package ggcorrplot using Spearman's Rank Correlation Coefficients at the *p* < 0.05 significance level. Results for ‘Gijnlim’ year 1 and year 2 were combined in a single matrix prior to analysis.

## 3 Results

### 3.1 CA regime affecting shelf-life extension

#### 3.1.1 Physiological measurements

Throughout cold storage in each of the different experiments, L* , C* and h° in the tips were similar between treatments. In contrast, the majority of colour differences between control and CA treatments occurred in the basal regions. Typically, chroma and lightness were highest in controls indicative of yellowing compared to all CA samples. Also, ‘Gijnlim’ spears in year 1 showed a significant drop in h° values in basal regions during shelf-life further indicating increased yellowing which was more pronounced for air stored (*ca*. 100) compared to CA stored spears (*ca*. 110) (**S3**).

Visual assessment of asparagus after boiling for 3 min, following removal from cold storage and after shelf-life, further confirmed significant colour differences between CA stored spears and control with the first retaining a darker green colour while air stored spears developed significant yellowing especially following shelf-life (**S4**). Another observation was that spears cooked immediately after removal from CA developed skin blistering, unlike spears stored under air, or spears subjected to shelf-life irrespective of treatment. The severity of blistering was positively correlated with CO_2_ concentration.

**Fig. 1:**
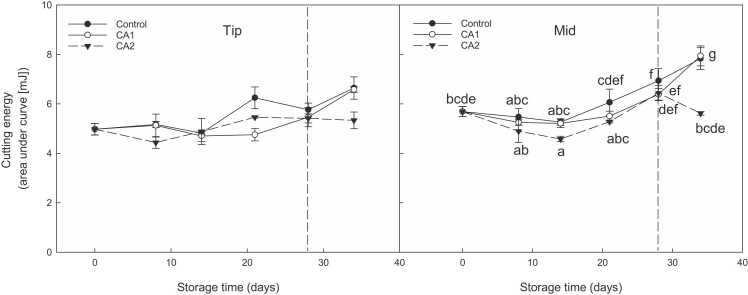
Effect of CA (CA1: 2.5 kPa O_2_ + 3 kPa CO_2_; CA2 – 2.5 kPa O_2_ + 6 kPa CO_2_) compared to control (air) on cutting energy (mJ) in tip and mid sections of asparagus spears (‘Gijnlim’) stored in 12 L boxes at 1 °C for 28 days (year 1). After cold storage (indicated by dashed line), spears were subjected to shelf-life (SL) assessment at 7 °C for 5 days. Multiple standard error bars are shown. Different letters denote significant differences.

CA treatments (CA1 [2.5 kPa O_2_ + 3 kPa CO_2_] and CA2 [2.5 kPa O_2_ + 6 kPa CO_2_]) assessed in year 1 maintained lower cutting energy towards the end of storage compared to air (Fig. 1). However, no clear statistical differences were observed in the second year.

In year 2, both CA treatments showed a delay in moisture loss (up to *ca.* 2%) during cold storage compared to control which reached up to 7% (**S5)**. This was also carried through into shelf-life samples where controls in the last storage week had significantly higher moisture loss (*ca.* 6.5%) compared to CA1 and CA2 (*ca.* 4–4.5%). In year 1, moisture loss was similar between treatments except at week 3 of cold storage where CA1 had significantly higher moisture loss (*ca.* 3%) compared to control and CA2 (*ca.* 1.6%).

Subjective assessment of the spears immediately after cold storage and at the end of shelf-life, revealed that both CA stored and control spears had no incidences of microbial spoilage or tip breakdown for up to two weeks in cold storage. After this point, there were incidences of mould, and tip breakdown, especially towards the end of cold storage; although this was generally significantly lower in CA stored spears compared to control. A similar pattern was observed for shelf-life, with CA stored spears having significantly lower incidences of mould and tip breakdown compared to control (**S6**). Some differences were also observed between tip breakdown incidence across different years and cultivars, with a higher incidence observed in year 1 compared to year 2 for ‘Gijnlim’, and ‘Jaleo’ showing overall the lowest rates of tip breakdown.

#### 3.1.2 Biochemical analysis

The results for individual sugars and asparagine measured in asparagus for the three experiments conducted over two successive years are presented in the Supplementary materials (**S7-S9**). Fructose and glucose rapidly depleted in the tips of ‘Gijnlim’ spears, during cold storage irrespective of treatment (year 1). Concentrations also gradually decreased in the basal regions, although CA stored spears (CA1 and CA2) maintained higher concentrations compared to control, (both during cold storage and shelf-life) with fructose maintaining levels similar to harvest (**S7**). A similar trend was observed the following year (year 2), although fructose and glucose concentrations at harvest were *ca.* 2-fold higher compared to the year before (**S8**). Furthermore, fructose and glucose levels were either stable or even increased during shelf-life, especially in the basal regions of CA stored spears. Comparison between the two CA regimes (CA2 and CA3) following cold storage and shelf life, showed no differences in fructose or glucose. However, during cold storage, basal regions in CA3 spears had significantly higher mean sucrose levels (*ca.* up to 1.2-fold) compared to air and CA2 (**S8**). An overall treatment effect was observed for ‘Jaleo’ during cold storage with total sugars in the tips being significantly higher in CA2 stored spears compared to control (**S9**).

‘Gijnlim’ spears from both years showed some variability in sucrose content during cold storage and shelf-life in both apical and basal regions, although the results were not statistically significant. Nevertheless, a clear pattern emerged where sucrose levels in the tips exhibited a significant increase (from between 1.6-fold to more than 3-fold) after seven days in shelf-life compared to the concentrations after removal from cold storage (**S7**, and **S8**). This trend was more pronounced in air stored spears compared to CA. The same trend was replicated for ‘Jaleo’ during shelf-life (**S9**).

**Fig. 2:**
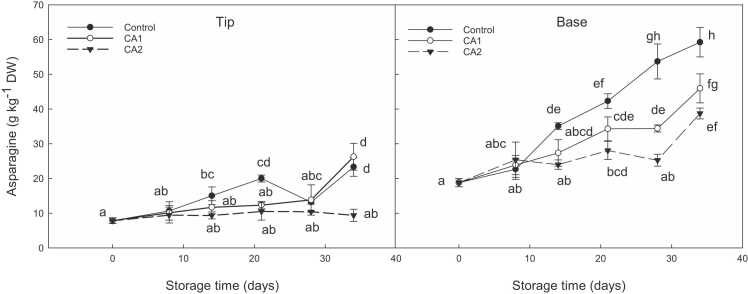
Effect of two CA (CA1: 2.5 kPa O_2_ + 3 kPa CO_2_; CA2 – 2.5 kPa O_2_ + 6 kPa CO_2_) treatments compared to control (air) on asparagine content (g kg^−1^ DW) in asparagus (‘Gijnlim’) during Exp. 1 cold storage (CS) within boxes and after shelf-life (SL) (day 34). Multiple standard error bars are shown. Different letters denote significant differences.

The amino acid L-asparagine was present at very low levels at harvest in both years, with concentrations following an upwards trend during storage, especially in the basal regions in aired stored samples in year 1 (Fig. 2**)**; no differences were noted between each respective CA regimes, with the exception of CA1 which reached the same levels as control during shelf-life in the tip region. Asparagine concentrations peaked in ‘Gijnlim’ during shelf-life in year 2 with tips having up to 2-fold higher asparagine levels in shelf-life samples compared to cold storage (**S8**). ‘Jaleo’ also exhibited a significant increase in asparagine concentrations in the tips during shelf-life, especially in control samples (**S9**).

**Fig. 3:**
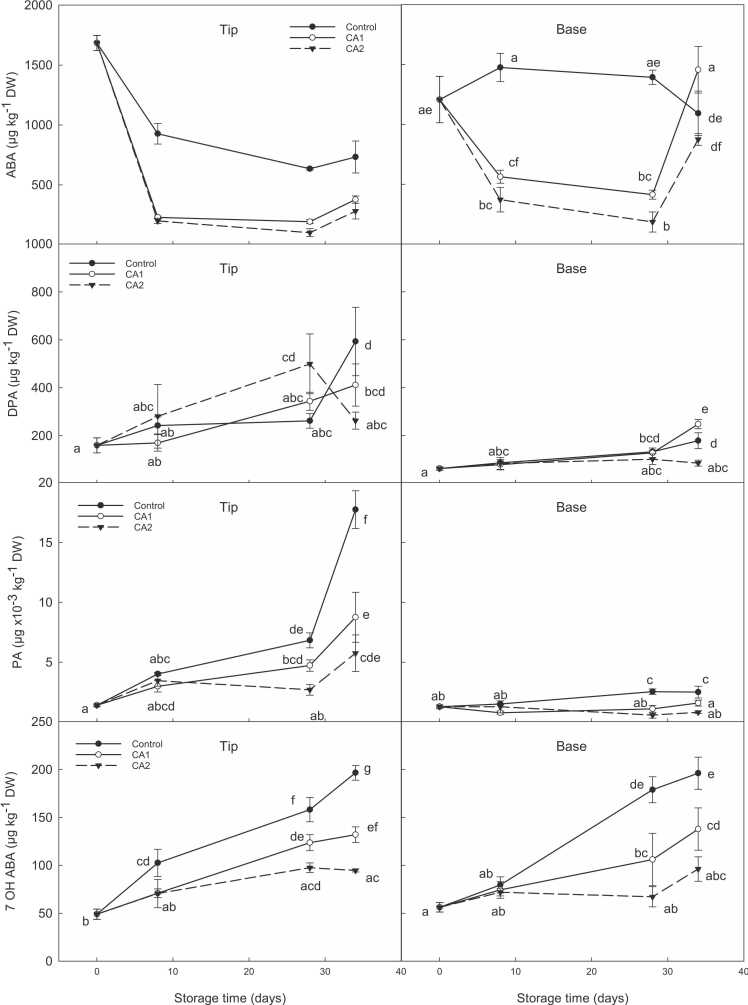
Effect of CA (CA1: 2.5 kPa O_2_ + 3 kPa CO_2_; CA2 – 2.5 kPa O_2_ + 6 kPa CO_2_) compared to control (air) on changes in ABA and ABA catabolites (*viz*. DPA, PA and 7-OH ABA) (µg kg^−1^ DW) in tips and basal regions of asparagus (‘Gijnlim’) during cold storage at 1 °C for 28 days within boxes (Exp. 1) followed by 7 days shelf-life assessment at 7 °C. Multiple standard error bars are shown. Different letters denote significant differences.

ABA concentrations were highest at harvest, but as cold storage progressed, levels in spears stored under all CA protocols rapidly decreased. In all experiments, ABA significantly dropped in both apical and basal regions of CA stored spears after just *ca.* 1 week of cold storage, with the largest decrease occurring during year 1 where ABA concentrations in the apical and basal regions decreased by *ca.* 87% and 60%, respectively (Fig. 3). ABA values remained significantly lower in CA stored spears compared to control throughout the duration of cold storage, with no significant differences between each respective CA regimes for each experiment.

A similar decreasing trend in ABA occurred in control tips, albeit less pronounced. Contrastingly, control basal regions exhibited a different trend to CA where ABA increased by up to *ca.* 1.2-fold and remained at an elevated level throughout cold storage. The ABA catabolites, phaseic acid (PA), dihydrophaseic acid (DPA) and 7′-hydroxy-ABA (7-OH-ABA) displayed a general increase during cold storage with values remaining overall lower in CA spears compared to control. PA, in particular, which represents the main catabolite of ABA in asparagus spears, exhibited a rapid increase in the tips of control samples, with values being 8-fold higher at the end of shelf-life compared to harvest, as opposed to a *ca.* 3-fold increase for CA stored spears (Exp. 1 Fig. 3). ABA catabolites were lowest in CA2 stored spears compared to CA1 with values for DPA (basal regions *ca.* 100 *vs.* 250 ng g^-1^ DW) and 7-OH-ABA (tips *ca.* 100 *vs.* 130 ng g^-1^ DW) significantly lower at the end of shelf life, respectively.

ABA concentrations also tended to increase during shelf-life after spears were removed from CA. ABA concentration in both the tips and basal regions of ‘Gijnlim’ spears stored under CA (CA1 and CA2) for 28 days, doubled after 7 days shelf-life (Fig. 3).

Finally, the results on ABA and ABA catabolites for asparagus spears stored using LabPods™ tended to mirror the observations for the boxes setup (**S10**).

### 3.2 Real time metabolic changes monitored using LabPods™

Storing asparagus within LabPods™ enabled metabolic measurements to be taken without disruption. Clear differences in respiration rate were observed between treatments with values overall significantly higher (*ca.* 2-fold) in the controls compared to samples stored under CA2 (2.5 kPa O_2_ + 6 kPa CO_2_). The RQ values for both air and CA stored spears remained below 1.0 for the duration of cold storage, but CA had an overall significantly lower mean value (mean *ca.* 0.74) compared to air stored spears (mean *ca*. 0.81) (**S11**). Colour changes followed a similar trend to that observed for spears stored in CA boxes. Furthermore, values for sugars (data not shown), ABA and ABA metabolites (**S10**) were in good agreement in both set ups.

### 3.3 ABA and ABA catabolites responsible for differentiating air *vs.* CA

**Fig. 4:**
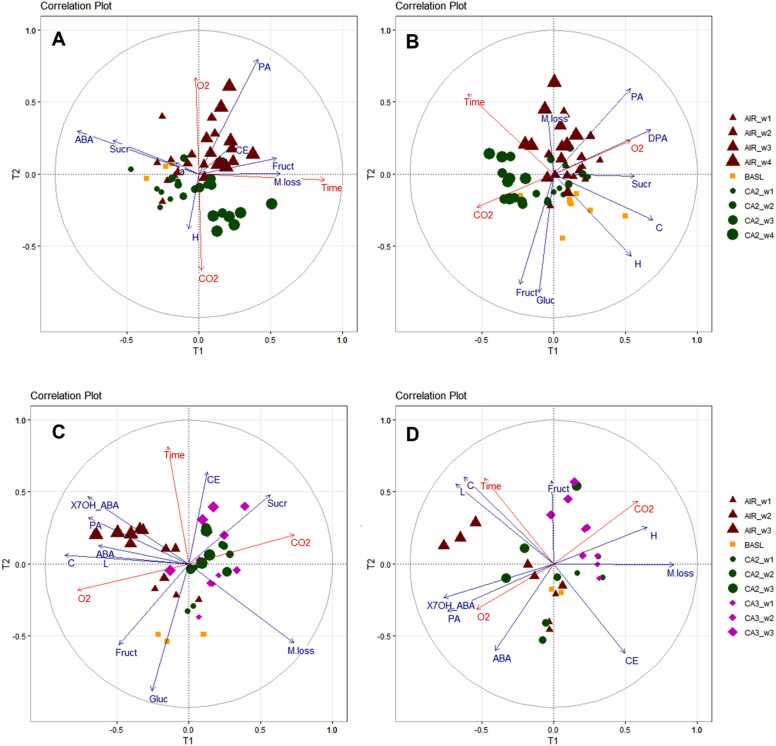
Correlation plots of ‘Jaleo’ asparagus spears (basal section) after 4 weeks of cold storage (A) followed by 1 week of shelf-life (B) (Exp. 3) and ‘Gijnlim’ spears (basal section) after 3 weeks of cold storage (C) followed by 1 week of shelf-life (D) (Exp. 2). The size of the triangles, circles and diamonds increases with storage time for air, CA2 and CA3 respectively. Squares represent samples measured at harvest (baseline). The blue arrows indicate prediction variables, and the red arrows indicate response variables. (BASL = baseline, AIR = air stored samples, CA2 = 2.5 kPa O_2_ + 6 kPa CO_2_, CA3 = 2.5 kPa O_2_ + 10 kPa CO_2_, w1 to w4 refers to number of weeks under cold storage. CE = cutting energy, M. loss = moisture loss, Fruct = fructose, Gluc = glucose, Sucr = sucrose, ABA = ABA, PA = PA, X7OH_ABA = 7-OH-ABA, H= hº, C= C*, L= L*).

To further investigate the correlation between the physiological and biochemical profile and the response variables, pls-r was conducted using data collected for ‘Gijnlim’ (year 2) and ‘Jaleo’ for which shelf-life assessment was performed each week. Fig. 4 shows the correlation plots created based on the pls-r results for the first two Latent Variables (LVs) for the basal regions of ‘Jaleo’ and ‘Gijnlim’ (year 2) during cold storage and subsequent shelf-life. A clear separation was observed between air and CA treatments during cold storage, especially after two weeks storage, with h° being the main variable driving the separation (Fig. 4A). In addition, basal sections were well separated based on storage time, with glucose and ABA driving the separation at the beginning of storage, while fructose and moisture loss were the variables contributing most to the separation towards the later stages. PA concentrations and cutting energy were positively correlated with high O_2_ concentrations and storage time and were the variables responsible for the separation of air stored samples from CA samples towards the end of storage. Apical sections also separated based on storage time, but the clusters were tighter implying a similar profile with the exception of week 1 (**S12**). When looking at the correlation plots for shelf-life, it was obvious that samples kept under CA were still well separated from air stored samples even after seven days under shelf-life conditions. Another deciding factor affecting the physiological and biochemical profile of asparagus spears was the time spent in cold storage prior to shelf-life as shown from the distribution of scores in the correlation plots. (Fig. 4B, D). Similar to what was observed in cold storage, PA and moisture loss at the end of shelf-life were positively associated with higher O_2_ concentrations and prolonged storage time during cold storage.

Correlation plots for ‘Gijnlim’ (Exp. 2) also revealed the diverse effects of gas concentrations and storage time on the physiological and biochemical profile of air *vs* CA stored spears. The scores showed a clear separation between air stored and CA stored spears during cold storage, for both basal (Fig. 4C) and apical sections (**S12C**). In contrast, scores corresponding to CA2 and CA3 (3 kPa O_2_+ 6 kPa CO_2_ and 3 kPa O_2_+ 10 kPa CO_2_, respectively) failed to form separate clusters based on different CO_2_ concentrations, but rather clustered according to storage time. By further examining the relative positions between the loadings and the response variables, as well as the individual graphs for each variable, we were able to gain more insight into the effect of storage conditions and time on asparagus quality. In basal sections the loadings for ABA and its catabolites PA and 7-OH-ABA were positively correlated with O_2_ concentrations and storage time, and the same was observed for C* and L* values. Moisture loss was negatively correlated with storage time and O_2_ concentrations, due to the fact that the absolute mass difference of air stored spears *vs* freshly harvested spears (baseline) increased proportionally to storage time. The loadings for the cutting energy in contrast, were positively correlated with storage time.

The separation of the scores according to gas concentrations and storage time was still evident after 7 days shelf-life, although the clusters were less tightly formed (Fig. 4D). Scores corresponding to CA3 (3 kPa O_2_ + 10 kPa) basal sections were further separated almost entirely from CA2 (2.5 kPa O_2_ + 6 kPa) scores with the latter clustering closer to scores for air stored spears. High O_2_ concentrations in air stored samples were strongly correlated with ABA catabolites, PA and 7-OH-ABA and negatively correlated with h°, while loadings for fructose were positively correlated with both storage time and CO_2_ concentrations.

Finally, we generated correlation matrices using Spearman's Rank Correlation Coefficients for each experiment which confirmed that during cold storage PA, DPA and 7-OH-ABA were in general positively correlated with asparagine, cutting energy, L* and moisture loss, and negatively correlated with sugars (**S13)**.

## 4 Discussion

### 4.1 Physiological changes under CA conditions

Parallel assessment with the LabPods™ and conventional CA (boxes) provided additional information of temporal changes in spear quality during cold storage. LabPods™ allowed produce to remain *in situ* whilst collecting accurate RR and RQ measurements, while the boxes set-up allowed for more frequent sampling. Throughout the duration of storage, RQ values remained ≤ 1 especially in CA stored spears (mean *ca.* 0.74) confirming that low O_2_ levels did not result in anaerobic metabolism at any point during storage. Similar changes in RQ have been reported by Hurst et al. (1994), where a drop in RQ (stabilising around *ca.* 0.75) occurred within the first 2 days of storage in air at 20 °C coinciding with a reduction (*ca*. 50%) in lipid content and an increase (3–4-fold) in malate synthase activity in asparagus tips. The results obtained from both set-ups showed similar trends confirming the reliability and reproducibility of the data obtained. To our knowledge, this is the first study to measure metabolic changes whilst asparagus spears remain *in situ* under CA during cold storage.

### 4.2 CA storage delayed senescence by preventing ABA and ABA catabolite accumulation

ABA and ABA catabolites were, for the first time, measured in asparagus during CA storage enabling greater insight into senescent processes, and comparisons between air and CA stored spears. Lower rates of respiration under cold storage were the main factor responsible for reduced moisture loss in spears irrespective of treatment and the coinciding drop in ABA, which is known to control stomata closure during deficit conditions and reduce transpiration water loss (Lim et al., 2015). Notably ABA had a slower decline in control compared to CA spears which could be attributed to higher RR.

A previous study in sweet cherries has also demonstrated a decrease in endogenous ABA content postharvest which was linked to a protective effect against over-ripening (Tijero et al., 2018). ABA is well documented to promote ripening and premature senescence both pre- and postharvest in other crops such as strawberries and cut flowers (Hunter et al., 2004, Chen et al., 2016). On the contrary delayed accumulation of ABA and suppression of ABA has been associated with an extension in storage life in crops such as strawberries (Bose et al., 2019). In asparagus we have previously shown that lower ABA concentrations at harvest were also correlated with better storability (Anastasiadi et al., 2020).

While ABA concentrations rapidly declined during cold storage in ‘Gijnlim’ year 1, PA concentrations exhibited the opposite trend with a more than 8-fold increase towards the end of cold storage and during shelf-life especially in the control samples, which coincided with rapid sugar depletion in the apical sections of the spears. A similar increase for PA concentrations was observed for ‘Gijnlim’ and ‘Jaleo’, in year 2 albeit less pronounced. Moreover, ABA and PA, were among the most important metabolites contributing to the separation of CA samples from air stored ones, even during shelf-life. Phytohormone activity was also tissue specific with PA increasing rapidly in the tips compared to the base. These results could indicate a sustained activation of the ABA biosynthetic path in response to dehydration as well as activation of ABA degradation processes in an effort to self-regulate the action of ABA and delay senescence. Indeed, ABA has long been thought to restrict its own accumulation by activating its catabolic enzymes (as reviewed by Xiong and Zhu, 2003). Based on these results and the strong correlation between PA and quality traits in asparagus (cutting energy, moisture, asparagine, individual sugar content), we propose that in addition to ABA, PA accumulation could also act as a biomarker of early senescence (air stored samples), with a more recent report suggesting PA retains biological activity similar to ABA and can selectively activate a subset of ABA receptors (Weng et al., 2016). These results confirm the important role of ABA and its catabolites in the retention of quality attributes of asparagus during storage.

### 4.3 Lower ABA and ABA catabolites prevented extensive sugar loss and asparagine accumulation under CA

Changes in sugar content during storage indicated that CA storage in general delayed senescence compared to the control, by maintaining overall higher sugar content in the tips and basal regions of asparagus spears, which is in good agreement with previous reports (Hurst et al., 1996, Renquist et al., 2005). Other studies have linked the sugar content in asparagus tips with tip breakdown susceptibility in asparagus cultivars (Lill et al., 1994), as well as with the combination of a decrease in sucrose in the tips, followed by an increase in asparagine (linked to senescence) (Renquist et al. 2005; Huyskens-Keil and Herppich, 2013). In this study, asparagine concentrations in the tips remained stable during cold storage under all CA regimes, but subsequently increased during shelf-life, although less than in control spears, which may explain the reduced tip breakdown incidence during cold storage under CA. Moreover, maintaining low asparagine levels in fresh produce could be a desirable trait following several reports showing a direct link between both dietary and endogenous asparagine and cancer metastasis (Knott et al., 2018), while asparagine is also a primary precursor of acrylamide a neurotoxin and potential carcinogen found in foods cooked at high temperatures (Lea et al., 2007).

Our data showed a strong negative correlation between ABA catabolites (PA and DPA in particular) and individual sugars, and a positive correlation with asparagine, in the apical part of the spears. Although the different sugar profile between CA and control samples can be partly explained by the lower RR under CA conditions, it is also possible that it could be influenced by a sugar-ABA crosstalk regulating senescence processes in asparagus. It has been proposed that ABA promotes sugar accumulation during active spear growth, however its role during storage is still not well understood. Moreover, the importance of PA in hormone crosstalk has only recently been recognised (Weng et al., 2016). Further research is required to elucidate the mechanisms under play between sugar and ABA biosynthetic and metabolic pathways.

### 4.4 Sugar profile change during shelf-life

A shift in sugar metabolism was observed during shelf-life with the total sugar content of asparagus spears increasing irrespective of storage regime. This increase was driven mainly by elevated sucrose concentrations in the tips of air stored spears, and fructose and glucose concentrations in the basal regions of CA stored spears. The increase in sucrose concentrations during shelf-life is in agreement with previous results (Anastasiadi et al., 2020) and has been hypothesised to be linked to a condensation reaction catalysed by sucrose phosphate synthase (SPS) as a result of variations in light and or temperature conditions. Studies in other crops such as lettuce and celery have also found that light exposure at 4 ºC and 7 ºC, respectively, had a protective effect on chlorophyll and promoted photosynthesis in fresh-cut produce resulting in sugar preservation or even increased sugar content (Zhan et al., 2013, Zhan et al., 2014).

The variation in individual sugar concentrations and profile observed both during cold storage and shelf-life could directly influence asparagus quality traits affecting sweetness perception.

### 4.5 CA helped maintain colour and textural quality

There have been a limited number of studies reporting the impact of CA storage on quality changes in asparagus, with the majority focused on high CO_2_ (*e.g.* 10 kPa) and low O_2_ (*e.g.* <5 kPa). The main quality indices affected by CA have included improved colour and reduced mould incidence (King et al., 1986, Lill and Corrigan, 1996, Renquist et al., 2005). In the present study and irrespective of gas composition, CA conditions did maintain darker more vibrant green spears by preventing ABA and ABA catabolite accumulation thus delaying senescence processes. Other studies exploring the use of CA/MAP technologies have also reported similar results where chlorophyll degradation is delayed thereby maintaining the green colour of asparagus (Renquist et al., 2005, Tenorio et al., 2004, Zhang et al., 2008, Li and Zhang, 2015, Techavuthiporn and Boonyaritthongchai, 2016).

Studies have so far failed to show any additional benefit of CA in retaining the textural properties of asparagus. White asparagus spears stored in CA (10 kPa CO_2_ + 17 kPa O_2_) at different temperatures, exhibited a loss of stiffness and an increase in fibrousness compared to air stored asparagus after 7 days (20 °C). High CO_2_ storage clearly inhibited the excessive postharvest accumulation of cellulose and hemicellulose (Huyskens-Keil and Herppich, 2013). In the present study, differences in cutting energy were observed between air stored spears and different CA regimes (CA1 and CA2) where CA was found to maintain lower values compared to control. Similar findings have been observed in asparagus stored under MAP (An et al., 2007, Zhang et al., 2008, Sothornvit and Kiatchanapaibul, 2009, Techavuthiporn and Boonyaritthongchai, 2016). The increase in cutting energy during storage was in agreement with our previous findings in air stored asparagus (Anastasiadi et al., 2020) and is indicative of lignification processes taking place which result in increased fibrousness and toughening of the spears. Furthermore, loadings for cutting energy were positively correlated with high O_2_ concentration and storage time in basal sections of ‘Jaleo’ spears during cold storage as well as with PA. Spearman's Rank Correlation analysis also showed strong positive correlation between PA and cutting energy (CE) both for apical and basal sections in ‘Gijnlim’ spears (year 1) during cold storage further supporting the hypothesis that high ABA and ABA catabolite levels accelerate senescence and quality deterioration. On the other hand, CE loadings in the pls-r correlation plot were negatively correlated with storage time during shelf-life for both tip and base in ‘Gijnlim’ spears (year 2), suggesting that prolonged CA did not convey any additional benefit in retaining textural characteristics for this cultivar compared to control.

The results from this study, confirm the positive effect of CA in maintaining quality parameters of green asparagus compared to control. Subjective evaluation showed that all CA regimes had a positive impact in reducing tip breakdown incidence, with some variability observed between years and different cultivars. ‘Jaleo’, had the lowest incidence of tip breakdown compared to ‘Gijnlim’ both during cold storage and shelf-life, which correlated with higher sugar content in the tips. Although we could not directly compare the different cultivars due to different harvest times, this result could indicate a link between genotype and tip breakdown susceptibility, as has previously been suggested (Lill et al., 1994). Nevertheless, although tip breakdown incidence was reduced under CA, it increased during shelf-life and proved to be the main limiting factor in successfully applying CA past two weeks.

### 4.6 CA prevented spear discolouration during cooking

CA stored spears retained a vibrant green colour after cooking even after 7 days of shelf-life, as opposed to control spears. It is however recommended that after removal from CA, asparagus spears are exposed to normal atmospheric conditions before distribution to reduce skin blistering susceptibility. It is hypothesised that excess CO_2_ is trapped underneath the epidermis forming small bubbles, which subsequently causes the skin to blister during cooking.

## 5 Conclusions

In the present study we demonstrated that low oxygen and high CO_2_ conditions significantly suppressed ABA and asparagine levels in asparagus during storage, coinciding with lower metabolic rates, higher sugars and more vibrant colour compared to control samples, thereby helping maintain asparagus quality for at least 2 weeks of cold storage followed by 7 days of shelf-life. The study revealed for the first time the influence of ABA and its catabolites on senescence processes in asparagus and could guide future efforts to further improve storage conditions and extend shelf-life. In addition, real-time monitoring of asparagus metabolic rates using LabPods™ could provide an early warning system for abiotic stress to better inform industry on “use-by” dates.

## CRediT authorship contribution statement

**Maria Anastasiadi:** Conceptualization, Methodology, Formal analysis, Software, Investigation, Visualization, Writing − original draft. **Emma Collings**: Conceptualization, Methodology, Formal analysis, Investigation, Visualization, Writing − original draft. **Leon Terry**: Conceptualization, Supervision, Funding acquisition, Writing − review & editing, Project administration.

## Declaration of Competing Interest

The authors declare that they have no known competing financial interests or personal relationships that could have appeared to influence the work reported in this paper.
