# Supplementary material for: Investigating the role of abscisic acid and its catabolites on senescence processes in green asparagus under controlled atmosphere (CA) storage regimes
Source: Postharvest Biol Technol. 2022 Jun;188:111892. doi: 10.1016/j.postharvbio.2022.111892 (PMC8994748; doi:10.1016/j.postharvbio.2022.111892)
Supplement: Supplementary file 1 — Supplementary material [file mmc1.docx]

**Investigating the role of abscisic acid and its catabolites on senescence processes in green asparagus under controlled atmosphere (CA) storage regimes**

**Supplementary material**

| 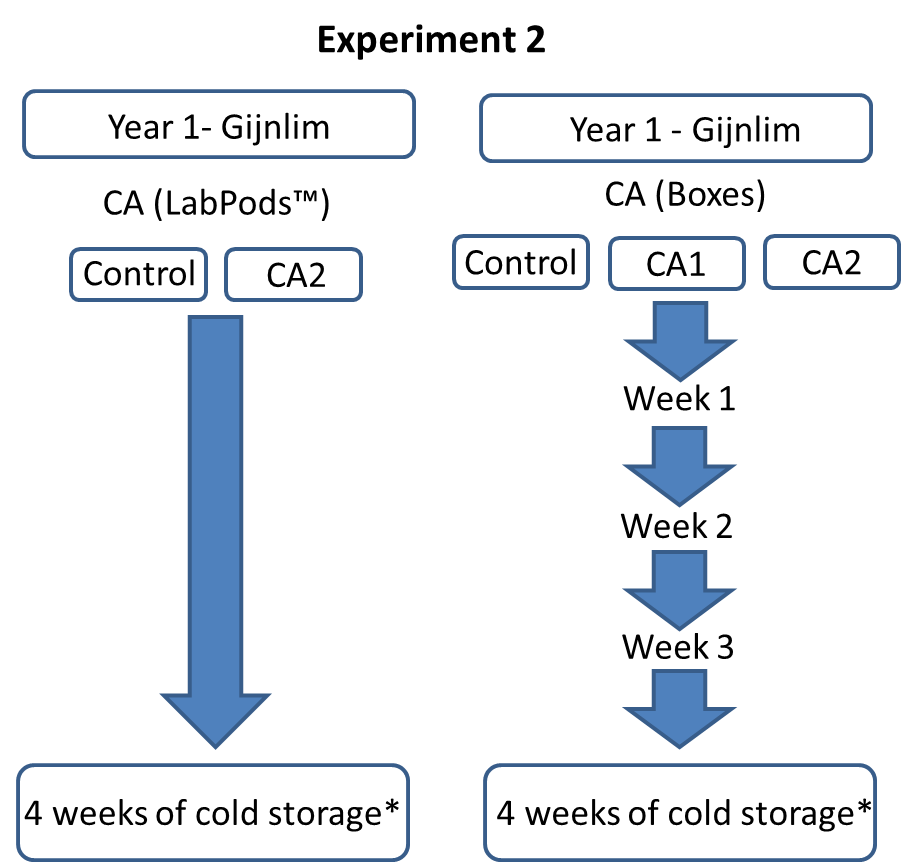 |
| --- |

| **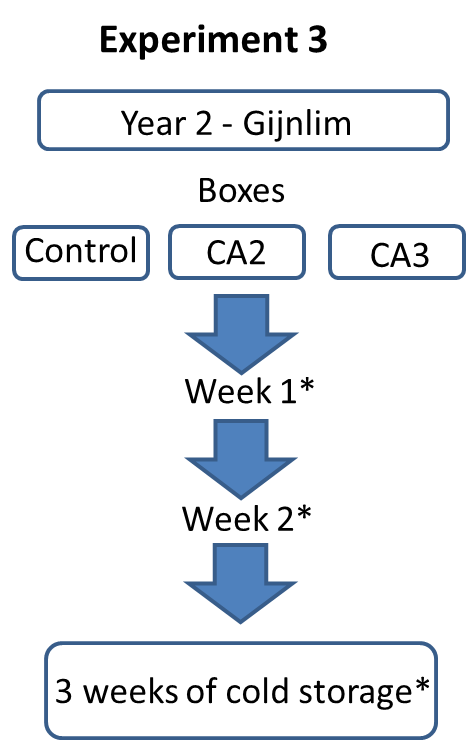** | **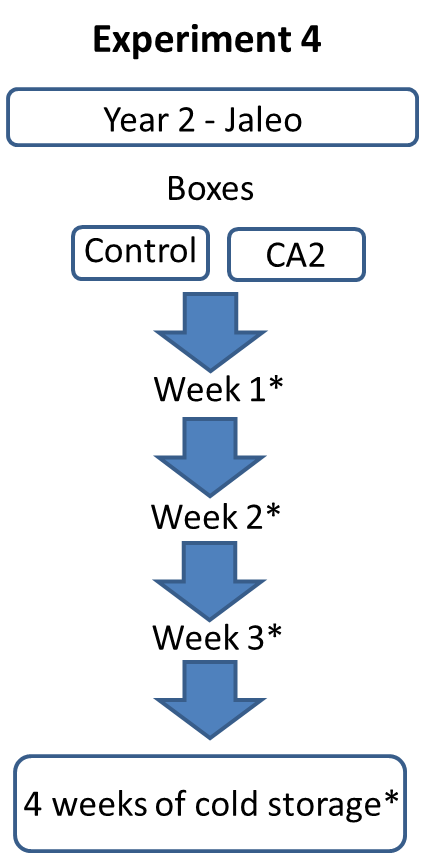** |
| --- | --- |

**S1**. Schematic of 3 experiments showing allocation of four batches of asparagus (‘Gijnlim’ and ‘Jaleo’) into 12 L boxes and LabPods opened weekly or after 4 weeks, respectively. Arrows indicate when spears were sampled for physiological and biochemical assessment. Treatments included: Air [control] 21 kPa O_2_ + 0.08 kPa CO_2_; Controlled Atmosphere [CA1] 2.5 kPa O_2_ + 3 kPa CO_2_; [CA2]: 2.5 kPa O_2_ + 6 kPa CO_2_; and [CA3] 2.5 kPa O_2_ + 10 kPa CO_2_. All treatments were subjected to cold storage (1 °C), followed by shelf-life assessment (marked with an *) within commercially packaged trays for 7 days at 7 °C.

**
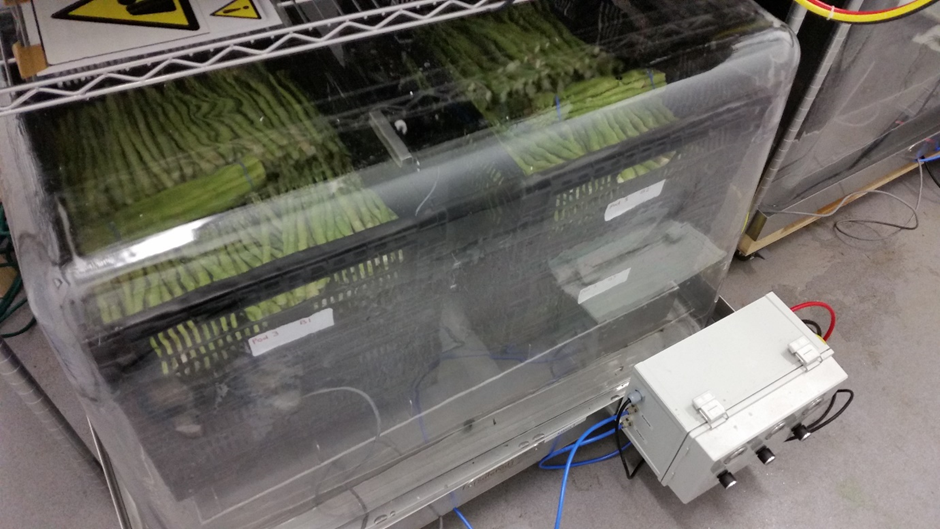
**

**S2.** Picture of the LabPods™ (Storage Control Systems Ltd, Kent UK).

B2

C1

C2

A1

A2

B1

**S3**, Effect of CA (CA1 – 2.5 kPa O_2_ + 3 kPa CO_2_ and CA2 – 2.5 kPa O_2_ + 6 kPa CO_2_) compared to control (air) on colour change ([A] lightness, [B] chroma, [C] hue angle) in [1] apical sections (tip) and [2] basal sections (base) of asparagus spears (‘Gijnlim’) stored in 12 L boxes at 1°C for 28 days followed by shelf-life assessment at 7°C for 5 days (Exp. 1). Standard error bars are shown. Different letters denote significant differences.

| **Control before** | **Control after** |
| --- | --- |
| 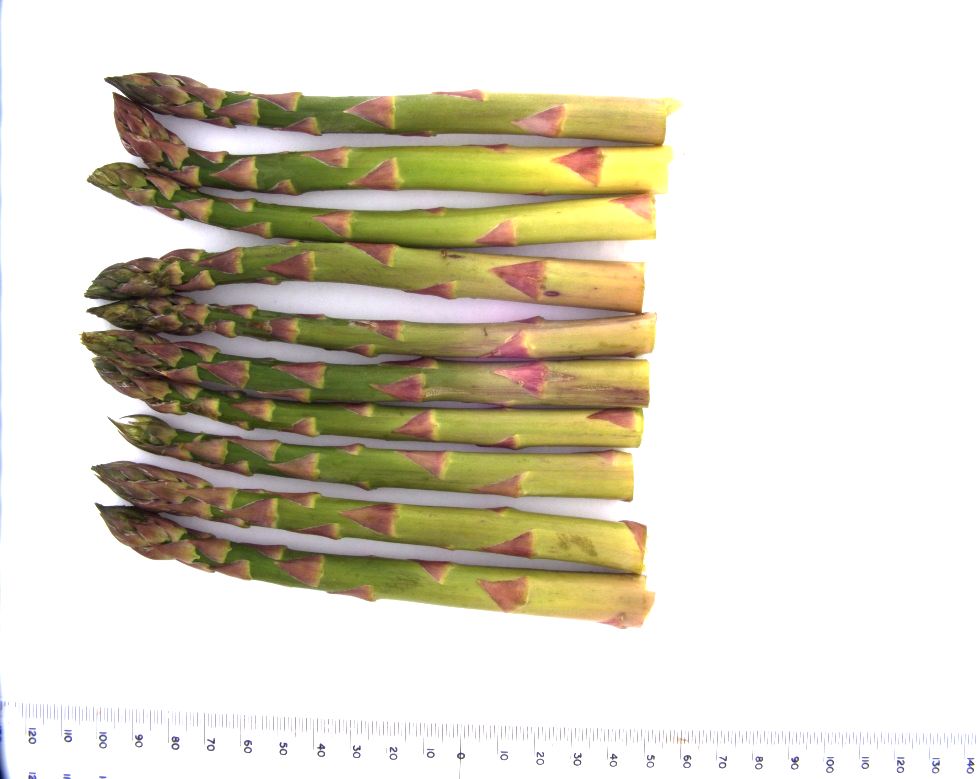 | 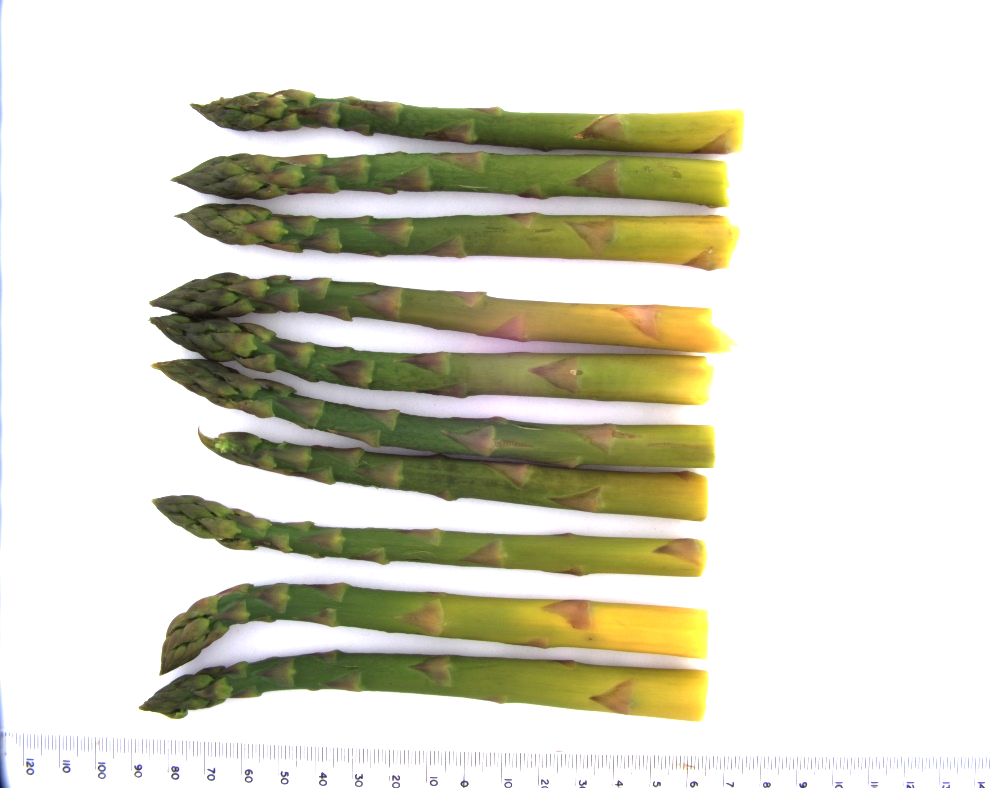 |
| **CA1 before** | **CA1 after** |
| 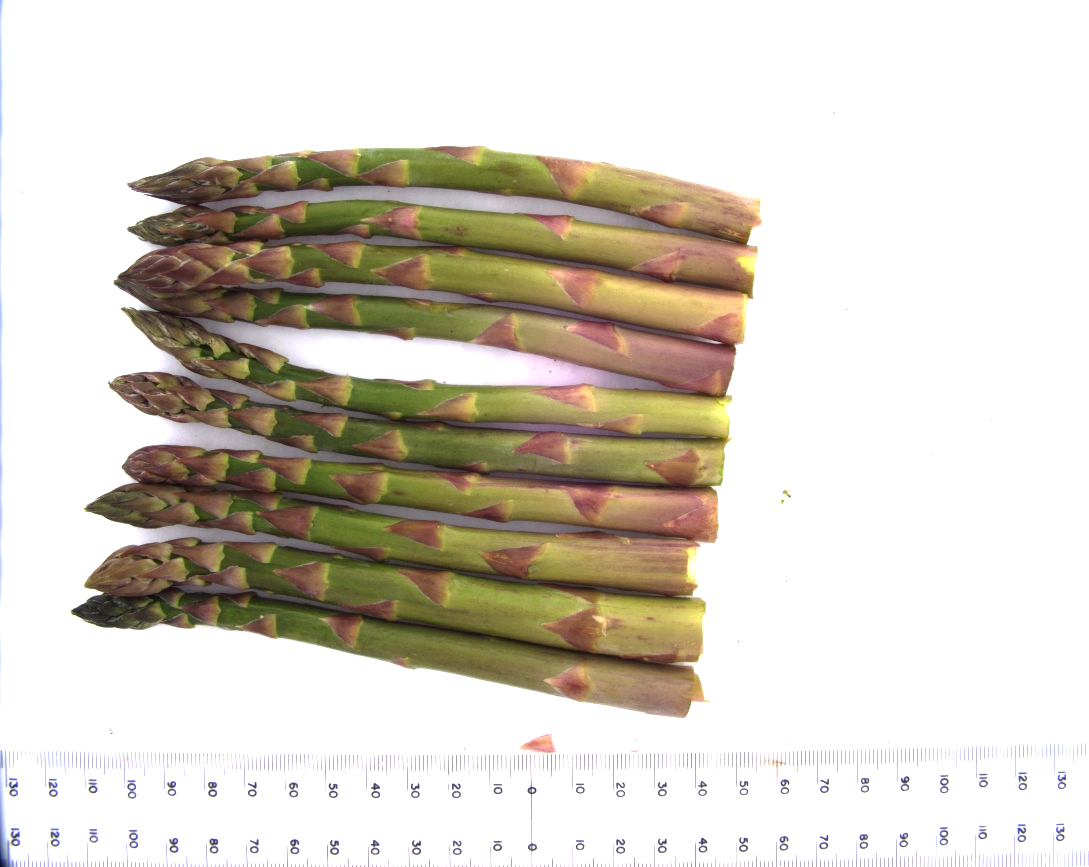 | 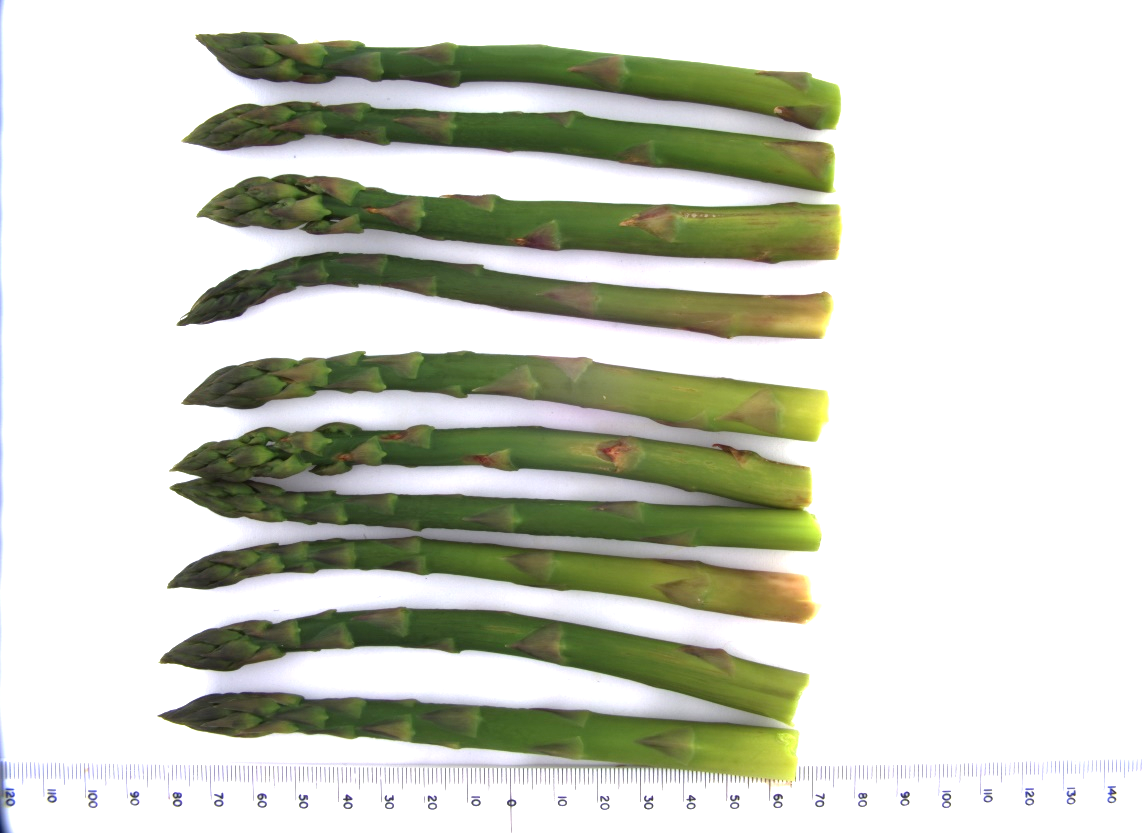 |

**S4**, Colour changes of spears cv. ‘Gijnlim’ before and after cooking following removal from cold storage under air or CA ([CA1: 2.5 kPa O_2_ + 3 kPa CO_2_] conditions (similar results for all CA conditions).

**S5,** Moisture loss data for each of the three experiments conducted over two successive years – numbers in italics are standard error, subscript letters denote significant differences

| **Experiment** | **Week** | **Treatment** | | | |
| --- | --- | --- | --- | --- | --- |
|  |  | Control | CA1 | CA2 | CA3 |
| 1 (boxes) | 1 | - | - | - |  |
|  | 2 | 1.85^ab^  *0.04* | 1.53^a^  *0.46* | 1.95^abc^  *0.18* |  |
|  | 3 | 1.38^a^  *0.31* | 3.20^d^  *0.08* | 1.70^ab^  *0.21* |  |
|  | 4 | 2.59^cd^  *0.41* | 2.62^cd^  *0.70* | 2.04^abc^  *0.08* |  |
|  | 5 | 2.33^bc^  *0.05* | 1.99^abc^  *0.14* | 2.02^abc^  *0.1* |  |
| 1 (LabPods^TM^) | 1 | 5.18  *2.75* |  | 3.83  *0.65* |  |
|  | 2 | 2.07  *0.32* |  | 1.72  *0.06* |  |
| 2 (boxes) | 1 | 1.76^ab^  *0.55* |  | 0.95^a^  *0.15* | 0.87^a^  *0.10* |
|  | 2 | 4.50^d^  *0.16* |  | 1.45^ab^  *0.17* | 1.36^ab^  *0.08* |
|  | 3 | 6.09^c^  *0.2* |  | 1.99^ab^  *0.61* | 2.0^ab^  *0.49* |
|  | 4 | 6.72^c^  *0.75* |  | 1.82^ab^  *0.59* | 2.27^b^  *0.09* |
|  | 0 SL | 4.95^a^  *0.12* |  |  |  |
|  | 1 SL | 4.0^ab^  *0.41* |  | 3.44^b^  *0.43* | 2.97^b^  *0.23* |
|  | 2 SL | 4.05^ab^  *0.7* |  | 4.71^a^  *0.43* | 3.81^ab^  *0.01* |
|  | 3 SL | 6.69^c^  *0.54* |  | 4.62^a^  *0.37* | 3.93^ab^  *0.12* |
| 3 (boxes) | 1 | 1.31a  *0.07* |  | 1.38ab  *0.04* |  |
|  | 2 | 1.13a  *0.11* |  | 1.02a  *0.15* |  |
|  | 3 | 2.67c  *0.32* |  | 1.58ab  *0.34* |  |
|  | 4 | 3.16c  *0.12* |  | 1.91bc  *0.11* |  |
|  | 0 baseline | 1.47  *0.08* |  |  |  |
|  | 1 SL | 1.60  *0.07* |  | 1.70  *0.06* |  |
|  | 2 SL | 2.08  *0.12* |  | 2.47  *0.15* |  |
|  | 3 SL | 2.26  *0.14* |  | 1.98  *0.21* |  |
|  | 4 SL | 2.10  *0.14* |  | 1.92  *0.07* |  |

**S6**, Subjective assessment data for the three experiments over 2 years. Tip rot (%) and Mould (%) represents the percentage of spears per treatment with tip rot and mould incidence respectively, after cold storage or shelf-life. Overall appearance represents a subjective score (per treatment) for freshness on a continuous scale from 0-5, with 5= fresh and 0= unacceptable. ND = no data, SL= shelf-life.

|  | **Treatment** | **Week** | **Tip rot (%)** | **Mould (%)** | **Overall appearance** |
| --- | --- | --- | --- | --- | --- |
| **Exp. 1** (‘Gijnlim’ year1) | Baseline | 0 | 0.0 | 0.0 | ND |
|  | Control | 1 | 0.0 | 0.0 | ND |
|  | CA1 | 1 | 0.0 | 0.0 | ND |
|  | CA2 | 1 | 0.0 | 0.0 | ND |
|  | Control | 2 | 0.0 | 0.0 | ND |
|  | CA1 | 2 | 0.0 | 0.0 | ND |
|  | CA2 | 2 | 0.0 | 0.0 | ND |
|  | Control | 3 | 67.0 | 16.0 | ND |
|  | CA1 | 3 | 29.0 | 11.0 | ND |
|  | CA2 | 3 | 27.0 | 0.0 | ND |
|  | Control | 4 | 68.0 | 3.0 | ND |
|  | CA1 | 4 | 54.0 | 0.0 | ND |
|  | CA2 | 4 | 43.0 | 0.0 | ND |
|  | Control | 4 SL | 100.0 | 100.0 | ND |
|  | CA1 | 4 SL | 72.0 | 21.0 | ND |
|  | CA2 | 4 SL | 83.0 | 17.0 | ND |
| **Exp. 2** (‘Gijnlim’ year2) | Baseline | 0 | 0.0 | 0.0 | 4.7 |
|  | Control | 1 | 0.0 | 0.0 | 4.0 |
|  | CA2 | 1 | 0.0 | 0.0 | 4.0 |
|  | CA3 | 1 | 0.0 | 0.0 | 4.0 |
|  | Control | 2 | 0.0 | 0.0 | 3.0 |
|  | CA2 | 2 | 0.0 | 0.0 | 3.8 |
|  | CA3 | 2 | 0.0 | 0.0 | 4.2 |
|  | Control | 3 | 43.1 | 0.3 | 1.0 |
|  | CA2 | 3 | 5.2 | 0.0 | 3.0 |
|  | CA3 | 3 | 3.2 | 0.0 | 3.0 |
|  | Control | 1 SL | 0.0 | 5.6 | 2.3 |
|  | CA2 | 1 SL | 0.0 | 0.0 | 3.0 |
|  | CA3 | 1 SL | 0.0 | 0.0 | 3.0 |
|  | Control | 2 SL | 55.5 | 33.3 | 1.2 |
|  | CA2 | 2 SL | 22.2^*^ | 0.0 | 2.7 |
|  | CA3 | 2 SL | 16.6^*^ | 11.1 | 2.0 |
|  | Control | 3 SL | 88.9 | 22.2 | 0.0 |
|  | CA2 | 3 SL | 44.4 | 0.0 | 1.0 |
|  | CA3 | 3 SL | 27.8 | 11.1 | 1.0 |
| **Exp. 3** (‘Jaleo’ year2) | BASELINE | 0 | 0.0 | 0.0 | 4.5 |
|  | Control | 1 | 0.0 | 0.0 | 4.3 |
|  | CA2 | 1 | 0.0 | 0.0 | 4.4 |
|  | Control | 2 | 0.0 | 0.0 | 4.2 |
|  | CA2 | 2 | 0.0 | 0.0 | 4.0 |
|  | Control | 3 | 5.6^*^ | 0.0 | 3.5 |
|  | CA2 | 3 | 11.1^*^ | 0.0 | 3.8 |
|  | Control | 4 | 25.0 | 4.4 | 1.0 |
|  | CA2 | 4 | 30.6 | 0.0 | 3.5 |
|  | Control | 1 SL | 0.0 | 0.0 | 4.1 |
|  | CA2 | 1 SL | 0.0 | 0.0 | 3.8 |
|  | Control | 2 SL | 0.0 | 11.1 | 1.0 |
|  | CA2 | 2 SL | 0.0 | 0.0 | 3.0 |
|  | Control | 3 SL | 1.8 | 5.6 | 1.5 |
|  | CA2 | 3 SL | 0.0 | 0.0 | 1.8 |
|  | Control | 4 SL | 0.7 | 11.1 | 1.0 |
|  | CA2 | 4 SL | 0.0 | 11.1 | 1.0 |

* early signs of tip rot (darkening)

**S7,** Effect of CA ([CA1: 2.5 kPa O_2_ + 3 kPa CO_2_] [CA2: 2.5 kPa O_2_ + 6 kPa CO_2_]) compared to control (air) on fructose, glucose, sucrose and asparagine concentration (g kg^-1^ DW) in the apical sections (tip) and basal sections (base) of asparagus spears (‘Gijnlim’) stored in 12 L boxes at 1°C for 28 days (Exp. 1). At the end of cold storage, spears were assessed under shelf-life (SL) conditions at 7 °C for 7 days (day 34). Data represent mean values (n=3). Standard error values shown in brackets.

| **Sugar** |  | **Tip** | | | **Base** | | |
| --- | --- | --- | --- | --- | --- | --- | --- |
|  | **Day** | **Control** | **CA1** | **CA2** | **Control** | **CA1** | **CA2** |
| Fructose | 0 | 13.82 | | | 144.14^d^ | | |
|  | 8 | 3.42 | 3.10 | 1.71 | 136.98^bcd^ | 134.26^bcd^ | 123.4^ab^ |
|  | 14 | - | - | - | 135.52^bcd^ | 146.72^d^ | 138.76^cd^ |
|  | 21 | - | - | - | 142.14^cd^ | 140.24^cd^ | 128.12^abc^ |
|  | 28 | - | - | - | 123.48^ab^ | 145.88^d^ | 143.04^d^ |
|  | 34 | - | - | - | 117.26^a^ | 141.98^cd^ | 135.6^bcd^ |
| Glucose | 0 | 6.98 | | | 107.3^e^ | | |
|  | 8 | - | - | - | 92.54^d^ | 92.74^d^ | 81.62^bcd^ |
|  | 14 | - | - | - | 76.92^bc^ | 91.88^d^ | 85.7^cd^ |
|  | 21 | - | - | - | 81.12^bcd^ | 87.02^cd^ | 77.24^bc^ |
|  | 28 | - | - | - | 70.26^b^ | 76.04^bc^ | 86.74^cd^ |
|  | 34 | - | - | - | 57.84^a^ | 79.18^bc^ | 71.36^b^ |
| Sucrose | 0 | 12.92 (2.6) | | | 23.62 (1.4) | | |
|  | 8 | 13.78 (1.5) | 13.34 (2.2) | 12.08 (2.0) | 57.04 (1.7) | 61.44 (5.1) | 67.26 (4.8) |
|  | 14 | 5.28 (2.8) | 8.32 (2.2) | 10.42 (2.1) | 58.42 (8.3) | 66.02 (7.0) | 66.92 (4.6) |
|  | 21 | 11.02 (0.3) | 9.88 (1.3) | 10.22 (3.4) | 54.54 (4.2) | 45.18 (7.7) | 57.34 (2.1) |
|  | 28 | 0.00 | 3.02 (3.0) | 7.24 (3.8) | 40.5 (2.5) | 45.64 (2.4) | 44.86 (2.9) |
|  | 34 | 10.58 (2.4) | 11.34 (2.2) | 5.42 (0.7) | 21.88 (4.6) | 22.14 (0.2) | 24.96 (2.5) |
| Asparagine | 0 | 7.88^a^ | | | 18.82^a^ | | |
|  | 8 | 10.68^ab^ | 10.14^ab^ | 9.48^ab^ | 22.68^ab^ | 23.88^ab^ | 25.42^abc^ |
|  | 14 | 15.06^bc^ | 11.7^ab^ | 9.40^ab^ | 35.08^de^ | 27.38^abcd^ | 24.04^ab^ |
|  | 21 | 20.00^cd^ | 12.34^ab^ | 10.52^ab^ | 42.32^ef^ | 34.32^cde^ | 28.1^bcd^ |
|  | 28 | 13.22^ab^ | 13.84^abc^ | 10.52^ab^ | 53.72^gh^ | 34.42^de^ | 25.30^ab^ |
|  | 34 | 23.34^d^ | 26.28^d^ | 9.42^ab^ | 59.24^h^ | 45.96^fg^ | 38.74^ef^ |
|  | 0 |  | 33.72 (4.5) |  |  | 275.06 (6.9) |  |
| Total sugars | 8 | 18.24 (3.7) | 18.64 (4.3) | 13.78 (3.0) | 286.56 (3.4) | 288.44 (10.6) | 272.28 (10.9) |
|  | 14 | 5.28 (2.8) | 8.32 (2.2) | 10.42 (2.1) | 270.86 (4.3) | 304.62 (6.5) | 291.38 (6.0) |
|  | 21 | 11.04 (0.3) | 9.88 (1.3) | 10.24 (3.4) | 277.8 (2.6) | 272.44 (15.5) | 262.7 (8.0) |
|  | 28 | 0.00 | 3.02 (3.0) | 7.24 (3.8) | 234.26 (7.0) | 280.3 (21.5) | 274.66 (14.1) |
|  | 34 | 10.60 (2.4) | 11.32 (2.2) | 5.42 (0.7) | 196.98 (12.7) | 246.22 (13.1) | 231.92 (8.7) |

Dashed line indicates none detected. Significant differences are denoted by different subscript letters within a column for each sugar compound. Values without letters indicate no significant differences were observed. Tip and basal regions were statistically analysed separately due to evident difference in values (values separated by vertical dashed line).

Purple = overall treatment effect

Green = overall day effect

Red = overall treatment and day effect

**S8,** Effect of CA ([CA2: 2.5 kPa O_2_ + 6 kPa CO_2_] [CA3: 2.5 kPa O_2_ + 10 kPa CO_2_]) compared to control (air) on fructose, glucose, sucrose concentration and asparagine (g kg^-1^ DW) in the apical sections (tip) and basal sections (base) of asparagus spears (‘Gijnlim’) cold stored (CS) in 12 L boxes at 1°C for 28 days (Exp. 2) followed by shelf-life (SL) assessment at 7 °C for 7 days. Data represent mean values (n=3). Standard error values shown in brackets.

| **CS** | **Tip** | | | | | | | | **Base** | | | | | | |
| --- | --- | --- | --- | --- | --- | --- | --- | --- | --- | --- | --- | --- | --- | --- | --- |
| **Week** | **Fructose** | | | | **Glucose** | | | | **Fructose** | | | **Glucose** | | | |
|  | **Control** | **CA2** | **CA3** | **Control** | | **CA2** | **CA3** | **Control** | | **CA2** | **CA3** | **Control** | **CA2** | **CA3** |  |
| 0 | 27.49 (3.1) | | | | 14.41 (1.1) | | | | 169.2 (3.9) | | | 143.45 (1.7) | | | |
| 1 | 12.54 (3.6) | 19.84 (2.6) | 9.87 (1.9) | 8.91 (1.4) | | 12.58 (0.9) | 12.27 (1.9) | 163.94 (4.3) | | 160.89 (3.6) | 160.72 (7.3) | 134.24 (0.4) | 138.18 (4.6) | 131.86 (9.9) |  |
| 2 | 4.33 (1.3) | 3.16 (0.4) | 5.89 (2.4) | 6.17 (0.4) | | 8.31 (0.4) | 9.85 (0.7) | 145.47 (9.6) | | 133.69 (3.3) | 149.87 (9.7) | 112.49 (9.1) | 104.05 (2.9) | 115.47 (9.5) |  |
| 3 | 3.45 (0.9) | 5.85 (0.1) | 3.96 (1.8) | 3.73 (0.2) | | 6.79 (1.3) | 7.93 (1.5) | 148.11 (3.7) | | 144.17 (2.9) | 131.94 (4.8) | 108.27 (4.3) | 108.7 (5.6) | 91.82 (5.5) |  |
| 4 | 2.11 (0.5) | 6.64 (1.9) | 2.20 (0.4) | 4.97 (1.2) | | 6.87 (1.1) | 6.74 (0.3) | 164.1 (9.6) | | 150.89 (4.3) | 152.99 (8.0) | 117.68 (3.5) | 107.49 (2.5) | 106.29 (12.0) |  |
| **SL** |  |  |  |  | |  |  |  | |  |  |  |  |  |  |
| 0 | 9.45^abc^ | | | | 9.65^ab^ | | | | 165.08 (12.4) | | | 129.12 (12.0) | | | |
| 1 | 8.29^abc^ | 4.18^a^ | 10.79^bc^ | 9.76^ab^ | | 6.23^ab^ | 8.61^ab^ | 142.66 (8.8) | | 154.48 (6.1) | 155.78 (4.0) | 104.48 (4.2) | 123.05 (8.2) | 120.67 (1.8) |  |
| 2 | 5.09^ab^ | 10.02^abc^ | 7.98^abc^ | 7.18^ab^ | | 14.89^c^ | 10.54^bc^ | 157.07 (10.0) | | 173.78 (5.3) | 190.72 (11.5) | 111.03 (2.9) | 121.8 (9.0) | 135.87 (8.2) |  |
| 3 | 11.31^c^ | 4.86^ab^ | 3.48^a^ | 8.46^ab^ | | 5.95^ab^ | 5.81^a^ | 159.75 (9.9) | | 183.34 (2.4) | 172.88 (4.2) | 121.11 (15.1) | 133.97 (7.0) | 123.7 (5.6) |  |

| **CS** | **Tip** | | | | | | **Base** | | | | | |
| --- | --- | --- | --- | --- | --- | --- | --- | --- | --- | --- | --- | --- |
|  | **Sucrose** | | | **Asparagine** | | | **Sucrose** | | | **Asparagine** | | |
| **Week** | **Control** | **CA2** | **CA3** | **Control** | **CA2** | **CA3** | **Control** | **CA2** | **CA3** | **Control** | **CA2** | **CA3** |
| 0 | 23.62 (1.79) | | | 14.16 (4.8) | | | 29.36 (2.6) | | | 23.60 (4.2) | | |
| 1 | 18.06 (8.40) | 20.38 (8.1) | 23.55 (1.40 | 15.82 (3.4) | 10.84 (2.9) | 21.98 (6.2) | 48.98 (7.7) | 34.57 (10.5) | 55.07 (0.6) | 16.64 (5.8) | 20.14 (2.9) | 19.93 (2.1) |
| 2 | 14.05 (5.3) | 28.08 (3.3) | 35.29 (6.2) | 18.31 (7.2) | 19.65 (5.0) | 11.63 (2.7) | 53.10 (7.2) | 62.89 (2.5) | 67.72 (4.5) | 20.41 (1.5) | 23.70 (3.1) | 20.85 (1.9) |
| 3 | 11.68 (1.5) | 24.51 (5.3) | 20.36 (1.8) | 21.87 (1.9) | 17.14 (2.0) | 13.33 (1.1) | 36.81 (1.8) | 50.53 (3.8) | 66.50 (4.0) | 26.78 (2.2) | 24.27 (1.6) | 27.26 (2.5) |
| 4 | 7.48 (2.0) | 20.96 (3.1) | 16.78 (0.9) | 21.68 (2.2) | 23.54 (2.7) | 13.81 (3.3) | 36.86 (1.7) | 59.58 (8.5) | 71.55 (13.2) | 29.35 (5.2) | 20.44 (3.0) | 23.79 (2.3) |
| **SL** |  |  |  |  |  |  |  |  |  |  |  |  |
| 0 | 27.91^abc^ | | | 32.43 (3.0) | | | 35.12 (5.6) | | | 28.15 (2.6) | | |
| 1 | 60.08^de^ | 22.29^bc^ | 30.86^abc^ | 20.14 (0.7) | 31.67 (3.1) | 29.92 (2.1) | 26.36 (4.8) | 26.36 (3.3) | 28.8 (4.0) | 26.61 (3.3) | 28.64 (3.4) | 29.33 (5.0) |
| 2 | 48.03^ade^ | 50.44^ade^ | 43.33^acd^ | 31.09 (5.4) | 27.95 (6.4) | 35.0 (2.1) | 31.11 (4.4) | 32.64 (4.06) | 34.73 (4.1) | 18.06 (1.2) | 25.87 (5.9) | 31.27 (2.1) |
| 3 | 70.67^e^ | 31.89abc | 16.18^b^ | 19.78 (0.6) | 35.14 (2.6) | 33.83 (2.6) | 45.82 (5.9) | 31.17 (3.9) | 37.06 (1.9) | 37.30 (12.3) | 27.49 (6.3) | 24.85 (4.2) |

Purple = overall treatment effect

Green = overall week effect

Red = overall treatment and week effect

**S9 -** Effect of CA2 (2.5 kPa O_2_ + 6 kPa CO_2_) compared to control (air) on fructose, glucose, sucrose and asparagine concentration (g kg^-1^ DW) in the apical sections (tip) and basal sections (base) of asparagus spears (‘Jaleo’) cold stored (CS) in 12 L boxes at 1°C for 26 days (Exp. 3) followed by shelf-life (SL) assessment at 7°C for 7 days. Data represent mean values (n=3). Standard error values shown in brackets.

| **CS** | **Tip** | | | |  | |  |  | |  | | **Base** | | | | |
| --- | --- | --- | --- | --- | --- | --- | --- | --- | --- | --- | --- | --- | --- | --- | --- | --- |
| **Week** | **Fructose** | | **Glucose** | | | **Sucrose** | | **Fructose** | | | **Glucose** | | | **Sucrose** | |  |
|  | **Control** | **CA2** | **Control** | **CA2** | | **Control** | **CA2** | **Control** | **CA2** | | **Control** | | **CA2** | **Control** | **CA2** |  |
| 0 | 38.52 (10.4) | | 13.14 (2.4) | | | 18.49 (4.8) | | 184.23 (5.7) | | | 162.82 (7.7) | | | 35.25 (1.1) | |  |
| 1 | 20.60 (2.7) | 21.48 (0.8) | 6.80 (1.3) | 9.18 (0.3) | | 41.0 (3.2) | 51.56 (6.1) | 175.0 (4.4) | 162.83 (3.3) | | 127.91 (7.3) | | 130.2 (8.5) | 61.46 (4.2) | 58.75 (0.2) |  |
| 2 | 17.18 (3.1) | 11.64 (0.2) | 5.76 (0.6) | 9.18 (0.5) | | 35.43 (7.4) | 36.83 (2.3) | 180.64 (9.7) | 164.70 (5.5) | | 120.37 (8.5) | | 117.45 (4.7) | 52.70 (1.6) | 54.36 (4.2) |  |
| 3 | 14.21 (1.4) | 20.09 (6.9) | 5.21 (0.9) | 10.01 (1.9) | | 35.13 (7.1) | 31.34 (3.5) | 181.36 (6.8) | 186.47 (5.5) | | 118.85 (8.0) | | 134.84 (4.1) | 51.26 (1.5) | 42.0 (2.1) |  |
| 4 | 13.30 (1.5) | 16.68 (0.3) | 4.66 (0.5) | 8.11 (0.5) | | 26.46 (4.3) | 23.73 (1.9) | 194.17 (8.4) | 183.49 (7.1) | | 133.29 (8.2) | | 143.07 (9.8) | 41.77 (4.3) | 31.10 (3.9) |  |
| **SL** |  |  |  |  | |  |  |  |  | |  | |  |  |  |  |
| 0 | 24.45 (5.4) | | 13.52 (3.7) | | | 67.98 (11.4) | | 185.60 (5.0) | | | 140.43 (5.1) | | | 32.69 (2.3) | |  |
| 1 | 27.28 (8.6) | 26.04 (1.9) | 14.50 (4.7) | 14.97 (2.1) | | 70.39 (14.6) | 82.00 (6.2) | 184.75 (11.5) | 182.88 (9.4) | | 130.67 (15.1) | | 126.35 (11.0) | 38.56 (1.8) | 28.04 (2.1) |  |
| 2 | 36.29 (3.6) | 18.10 (3.1) | 23.61 (3.3) | 10.32 (3.0) | | 96.32 (12.5) | 57.6 (9.0) | 181.66 (5.4) | 188.07 (5.4) | | 117.89 (2.8) | | 122.19 (3.7) | 40.16 (2.3) | 33.54 (3.2) |  |
| 3 | 18.85 (5.9) | 14.71 (1.8) | 11.07 (3.3) | 6.36 (0.9) | | 62.86 (10.5) | 37.08 (5.3) | 170.98 (17.2) | 205.37 (5.9) | | 107.22 (15.4) | | 134.15 (2.6) | 26.41 (2.0) | 20.16 (2.1) |  |
| 4 | 13.23 (2.4) | 16.37 (5.9) | 7.81 (1.2) | 7.31 (2.4) | | 50.16 (8.24) | 36.28 (3.0) | 166.29 (5.3) | 193.36 (8.1) | | 100.79 (7.4) | | 131.19 (8.1) | 29.98 (0.4) | 24.11 (4.2) |  |

| **CS** | **Tip** | | **Base** | |
| --- | --- | --- | --- | --- |
|  | **Asparagine** | | **Asparagine** | |
| **Week** | **Control** | **CA2** | **Control** | **CA2** |
| 0 | 5.59 (1.2) | | 21.15 (2.4) | |
| 1 | 6.83 (0.8) | 5.29 (0.1) | 23.49 (2.2) | 19.72 (1.6) |
| 2 | 10.61 (2.0) | 8.35 (0.8) | 26.79 (4.8) | 28.31 (0.8) |
| 3 | 11.98 (0.7) | 5.93 (0.6) | 24.02 (2.7) | 23.19 (1.8) |
| 4 | 14.13 (2.1) | 7.26 (1.1) | 32.03 (6.2) | 23.72 (4.2) |
| **SL** |  |  |  |  |
| 0 | 31.12 ^ab^ | | 32.98 (5.8) | |
| 1 | 42.33 ^ac^ | 30.53 ^ab^ | 27.25 (3.5) | 28.16 (5.2) |
| 2 | 31.33 ^ab^ | 45.52 ^c^ | 33.26 (1.6) | 37.42 (5.8) |
| 3 | 40.52 ^ac^ | 32.74 ^ab^ | 40.78 (1.9) | 29.25 (4.7) |
| 4 | 36.94 ^ac^ | 21.22 ^b^ | 40.07 (6.6) | 24.07 (3.2) |

Purple = overall treatment effect

Green = overall week effect

Red = overall treatment and week effect

**S10** - Effect of CA (CA2 – 2.5 kPa O_2_ + 6 kPa CO_2_) compared to control (air) on changes in ABA and ABA catabolites (*viz*. ABA, DPA, PA and 7-OH ABA) (µg kg^-1^ DW) in tips and basal regions of asparagus (‘Gijnlim’) during cold storage at 1°C for 28 days within Labpods™ (Exp. 1) followed by 7 days shelf-life assessment at 7 °C. Numbers in italics represent standard error. Different letters denote significant differences.

| **Spatial region** | **ABA & ABA catabolites** | **Sampling day** | **Control** | **CA2** |
| --- | --- | --- | --- | --- |
| Tips | ABA  µg kg^-1^ DW | 0 | 1684.16^d^  *63.25* | |
|  |  | 28 | 686.92^a^  *58.20* | 116.0^b^  *18.87* |
|  |  | 34 | 579.0^a^  *62.30* | 304.42^c^  *28.64* |
|  | DPA  µg kg^-1^ DW | 0 | 159.39  *31.44* | |
|  |  | 28 | 318.40  *46.53* | 217.94  *26.26* |
|  |  | 34 | 425.50  *44.01* | 279.24  *33.65* |
|  | PA  µg x10^-3^ kg^-1^ DW | 0 | 1.40  *0.11* | |
|  |  | 28 | 4.73  *0.33* | 1.97  *0.35* |
|  |  | 34 | 4.68  *0.47* | 2.19  *0.30* |
|  | 7 OH ABA  µg kg^-1^ DW | 0 | 48.93^a^  *5.35* | |
|  |  | 28 | 155.76^b^  *12.84* | 85.49^a^  *14.18* |
|  |  | 34 | 193.53^c^  *10.82* | 64.89^a^  *4.44* |
| Basal | ABA  µg kg^-1^ DW | 0 | 1207.54^a^  *193.51* | |
|  |  | 28 | 1235.84^a^  *109.23* | 223.92^b^  *22.74* |
|  |  | 34 | 1184.35^a^  *74.29* | 610.12^c^  *36.52* |
|  | DPA  µg kg^-1^ DW | 0 | 62.76  *6.84* | |
|  |  | 28 | 150.92  *20.12* | 56.35  *6.26* |
|  |  | 34 | 188.56  *26.95* | 83.96  *14.48* |
|  | PA  µg x10^-3^ kg^-1^ DW | 0 | 1.28  *0.14* | |
|  |  | 28 | 1.16  *0.19* | 0.37  *0.08* |
|  |  | 34 | 1.21  *0.17* | 0.50  *0.09* |
|  | 7-OH ABA  µg kg^-1^ DW | 0 | 56.18^a^  *4.93* | |
|  |  | 28 | 103.67^b^  *9.84* | 40.95^a^  *3.26* |
|  |  | 34 | 148.57^c^  *12.01* | 45.74^a^  *3.35* |

B.

A.

**S11,** Effect of low oxygen (CA2: 2.5 kPa O_2_ + 6 kPa CO_2_) compared to control (air) on [A] respiration rate (nmols kg^-1^ s^-1^) and [B] RQ in asparagus (‘Gijnlim’) during storage within LabPods™ at 1°C for 4 weeks (Exp. 1). Standard error bars are shown.

**
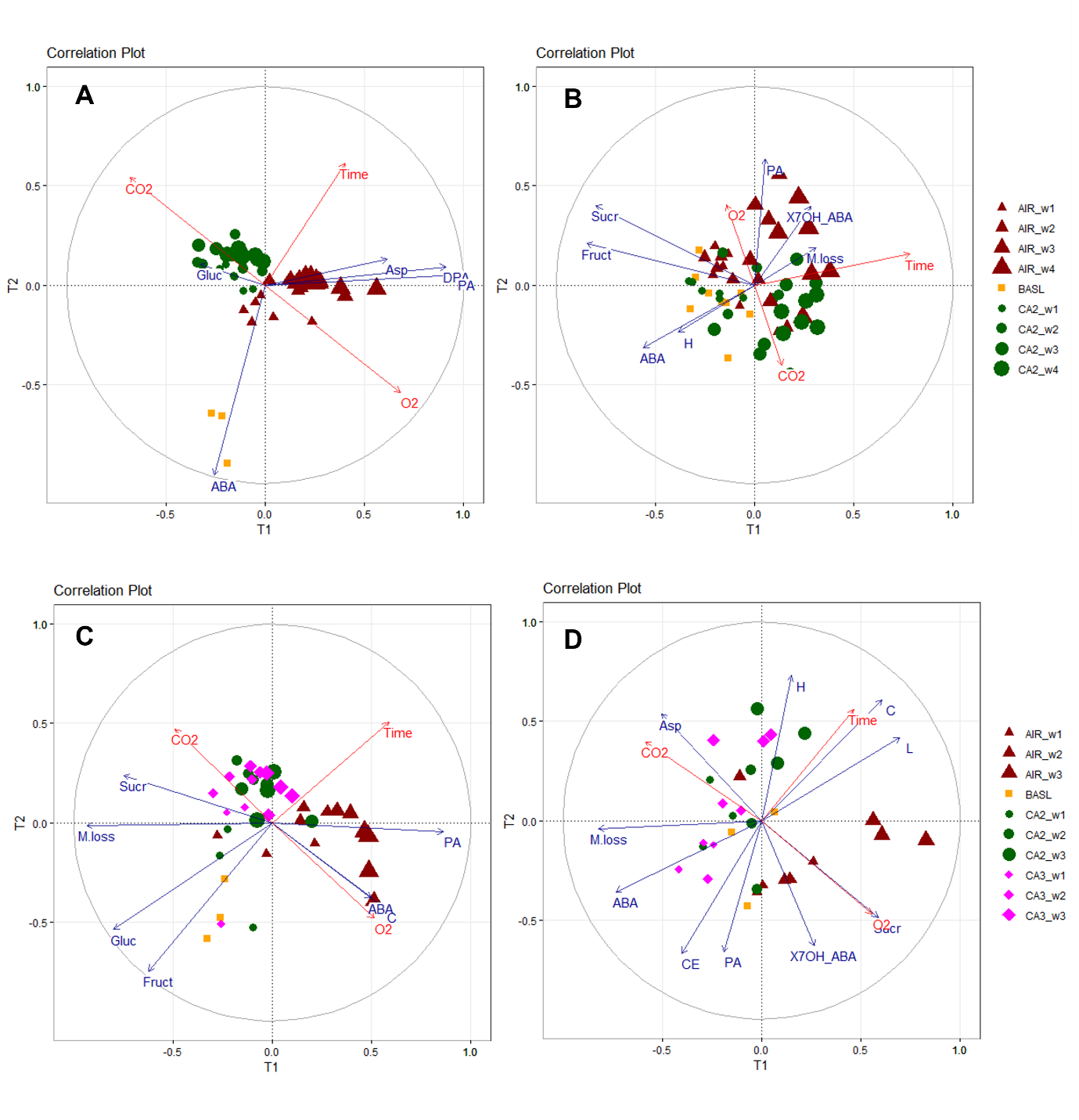
**

**S12,** Correlation plots of ‘Jaleo’ spear apical sections (tips) after 4 weeks of cold storage (A) followed by 1 week of shelf-life (B) (Exp. 3 and ‘Gijnlim’ spears apical sections (tip) after 3 weeks of cold storage (C) followed by 1 week of shelf-life (D) (Exp. 2). The size of the triangles, circles and diamonds increases with storage time for air, CA2 and CA3 respectively. Squares represent samples measured at harvest (baseline). The blue arrows indicate prediction variables and the red arrows indicate response variables. (BASL = baseline, AIR = air stored samples, CA2 = 2.5 kPa O_2_ + 6 kPa CO_2_, CA3 = 2.5 kPa O_2_ + 10 kPa CO_2_, w1 to w4 refers to number of weeks under cold storage. CE = cutting energy, M. loss = moisture loss, Asp = asparagine, Fruct = fructose, Gluc = glucose, Sucr = sucrose, ABA = ABA, PA = PA, X7OH_ABA = 7-OH-ABA, H= hº, C= C*, L= L*).


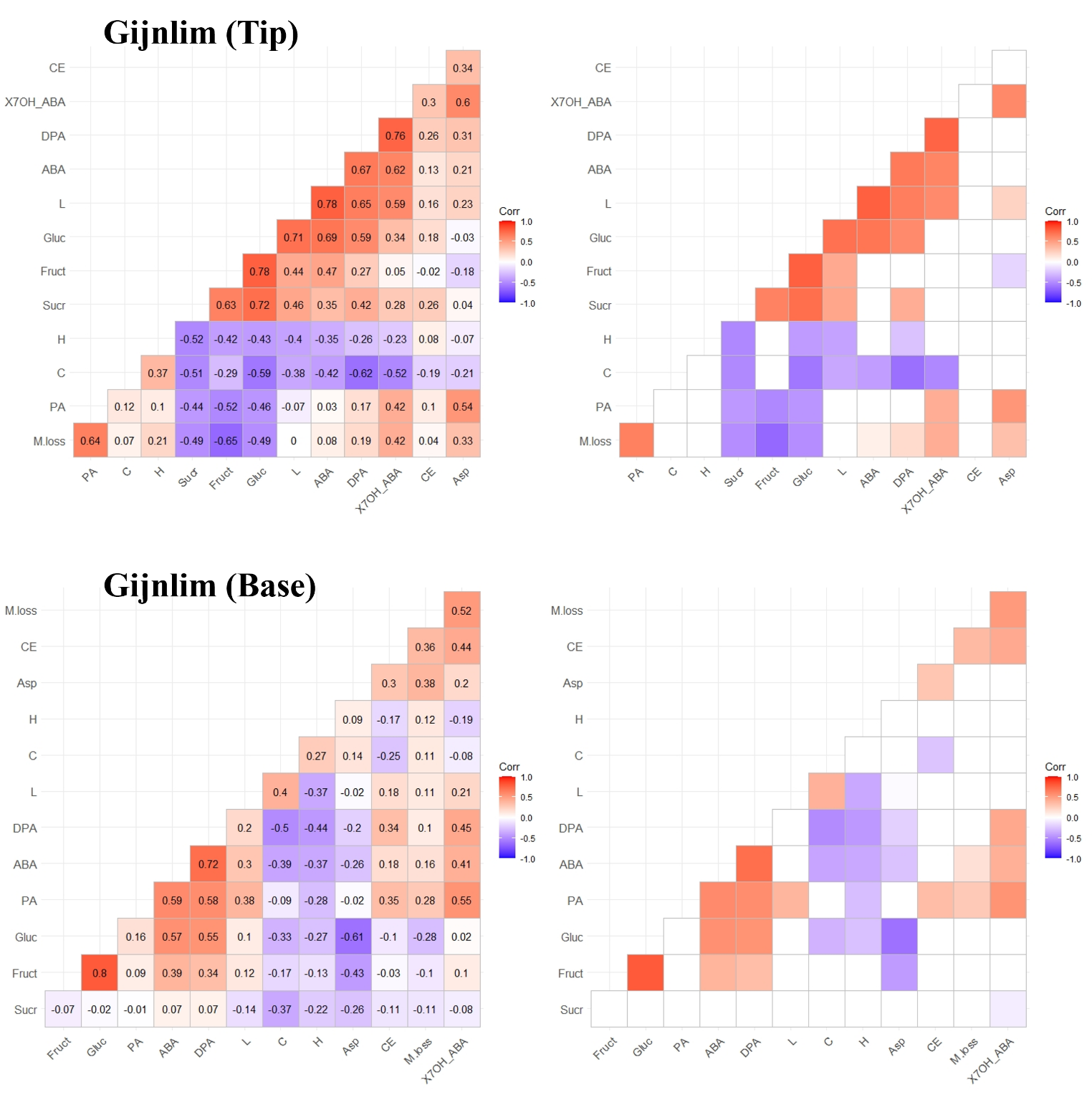


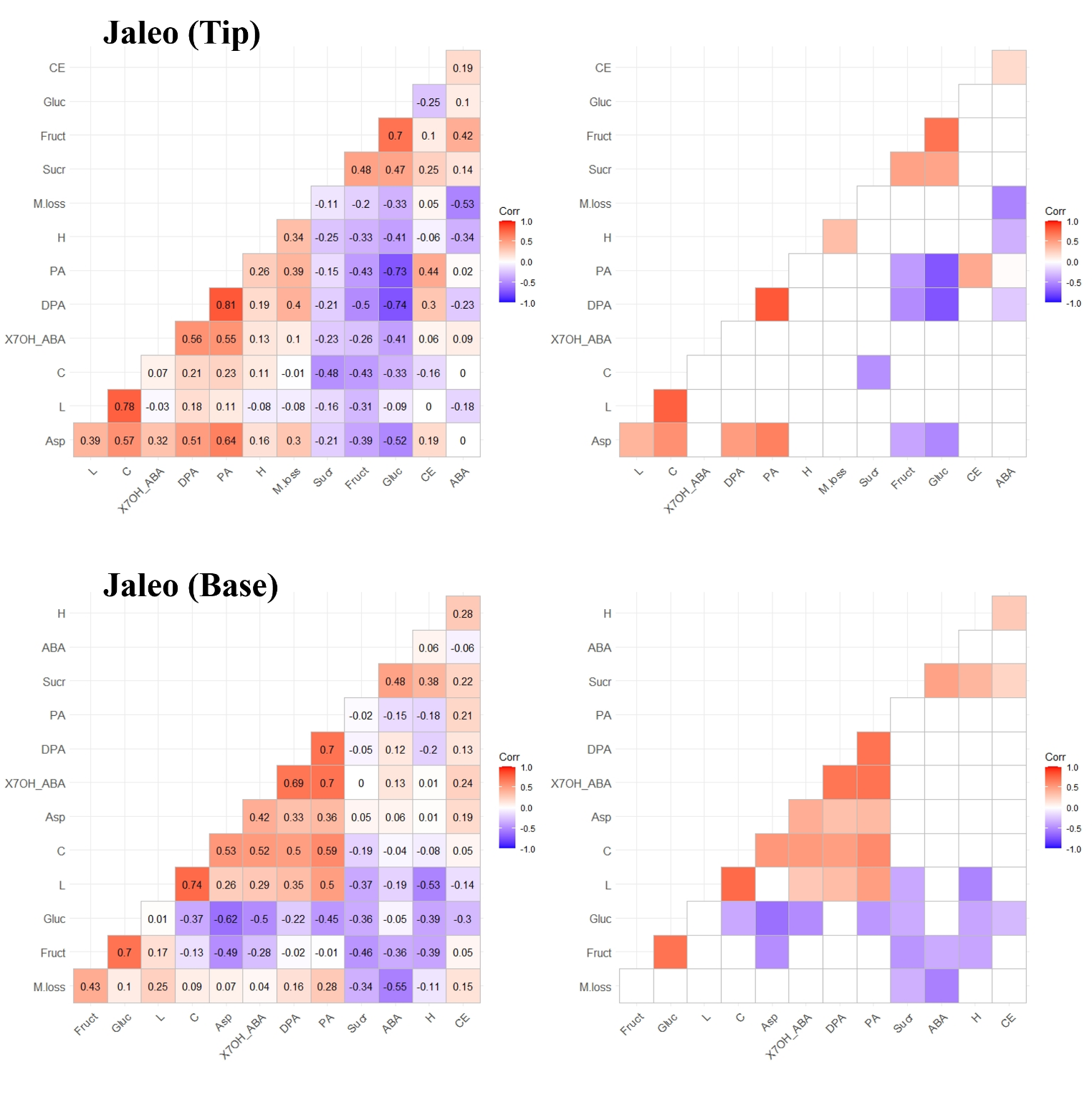


**S13**, Corelation heatmaps for physiological and biochemical parameters for ‘Gijnlim’ (year 1 and 2 combined) and ‘Jaleo’ under cold storage. The heatmaps on the left show Spearman’s Rank Correlation Coefficients for each parameter combination, while the heatmap on the right shows only statistically significant coefficients (*p<0.05*).
